# Chiroptical properties of 1,3-diphenylallene-anchored tetrathiafulvalene and its polymer synthesis

**DOI:** 10.3762/bjoc.11.109

**Published:** 2015-06-08

**Authors:** Masashi Hasegawa, Junta Endo, Seiya Iwata, Toshiaki Shimasaki, Yasuhiro Mazaki

**Affiliations:** 1 Department of Chemistry, School of Science, Kitasato University, 1-15-1 Kitasato, Minami-ku, Sagamihara, Kanagawa 252-0373, Japan; 2 Graduate School of Engineering, Chiba Institute of Technology, 2-17-1 Tsudanuma, Narashino, Chiba 275-0016, Japan

**Keywords:** allene, axial chirality, chiroptical properties, redox, tetrathiafulvalene

## Abstract

A novel tetrathiafulvalene dimer, bridged by a chiral 1,3-diphenylallene framework, has been prepared as an optically active compound having strong chiroptical properties. Although a chiral allene bearing strong electron-donating group(s) often undergoes slow photoracemization even in daylight, the present allene is totally configurationally stable under ordinary conditions. Each isomer possesses pronounced chiroptical properties in its ECD spectra reflecting the chiral allene framework. Moreover, the elongation of the chiral main chain was also carried out by direct C–H activation of the TTF unit, and the chiroptical properties of the resulting polymer were also investigated.

## Introduction

Recently, there has been a growing interest in chiral π-conjugated systems having strong chiroptical properties because they have great potential for use in optical devices involving polarized light [1–3]. Chiral response, in electronic circular dichroism (ECD) based on an exciton coupling between two adequate π-chromophores, can express a pronounced chiroptical effect over various optical energy regions. Therefore, embedding the chromophores into a chiral rigid framework can be a practical molecular design for ascertaining chiroptical materials [4–7].

**Figure 1:**
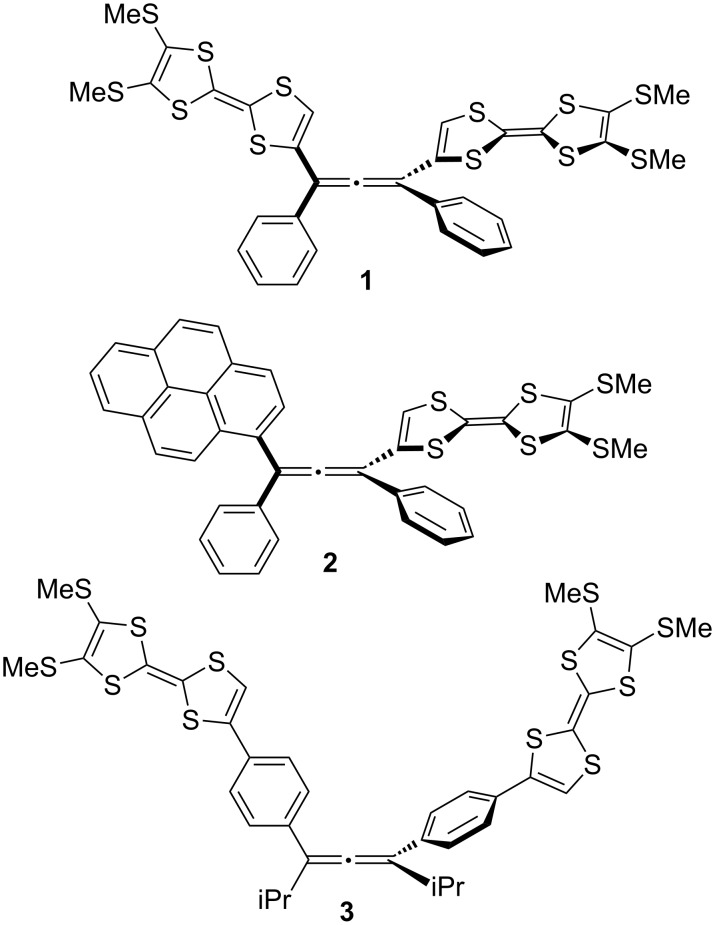
TTF-substituted allenes **1**–**3**.

A symmetric allene framework is one of the most reliable chiral resources that can preserve a consistent orientation of the chromophores [8–9]. We recently introduced 1,3-bis(tetrathiafulvalenyl)allene derivatives **1**, as a new class of chiral electrochromic (EC) materials consisting of redox-active chromophores and a non-centrochiral framework (Figure 1) [10]. The intensive Cotton effect on the ECD spectra is switchable by tuning the electronic structure of the tetrathiafulvalene (TTF) moieties. However, compound **1** exhibited slow racemization in solution under daylight. The chirality of an allene is configurationally firm in general, because the barrier of the rotation of the allenic double bonds is quite high (Δ*G*^‡^ = 180 kJ mol^−1^ for CH_3_CH=C=CHCH_3_) [11]. In contrast, several allenes directly connected with electron-donating groups occasionally underwent photoracemization [12–13]. Although the mechanism of the photoracemization is not clear, a comparative examination of the racemization rate in **1** and **2**, the latter of which is a dissymmetric allene having a TTF and a pyrenyl group at 1,3-position, suggests that the direct connection of the TTF units may strongly affect the fast racemization [14]. From this point of view, we decided to employ a 1,3-diphenylallene derivative (**3**) as a stable chiral framework. We conceive that the insertion of the phenylene units between the central chiral allene and TTFs would overcome the vulnerability toward the photoracemization under ambient conditions. Previsously, Krause and co-workers reported the first synthesis of a cyclic allenophane involving a racemic 1,3-diphenyl allene unit [15]. In addition, Fallis and co-workers synthesized a cyclic oligoallene based on a chiral 1,3-diphenylallene [16]. More recently, Kijima and co-workers reported the use of a racemic 1,3-diphenylallene framework as a new building block for a π-conjugated polymer [17]. However, there have been few investigations regarding the use of 1,3-diphenylallene as a chiral source and involving the detailed investigations of the chiroptical property. In this paper, we report the synthesis of chiral 1,3-bis(4-(tetrathiafulvalenenyl)phenyl)allene derivative **3** and its chiroptical properties. Furthermore, the first synthesis of a chiral copolymer containing TTFs based on a chiral allenic framework (poly-**3**: PTDPA is also demonstrated.

## Results and Discussion

**Scheme 1:**
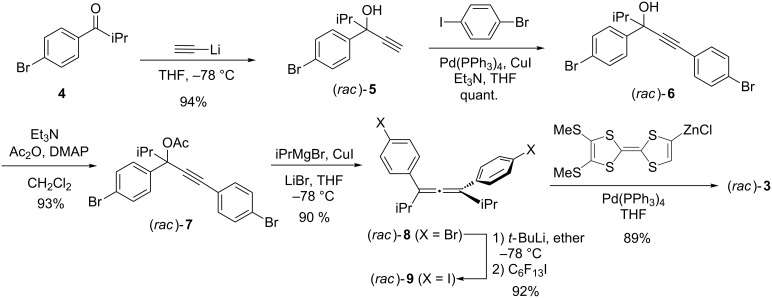
Synthesis of **3**.

To anchor the TTF groups into the allene framework, we chose a cross-coupling reaction of unknown 1,3-bis(4-iodophenyl)-1,3-diisopropylallene (**9**) with a TTF-zinc intermediate (Scheme 1). Previously, 1,3-bis(2-bromophenyl)allene derivatives were reported by Ready et al. as a precursor of an asymmetric catalyst [18]. To obtain the chiral 1,3-bis(4-bromophenyl)allene (**8**) in a larger scale, we modified the synthesis and the chiral separation process as shown in Scheme 1. Thus, starting with 1-(4-bromophenyl)-2-methylpropanone (**4**), which was prepared from bromobenzaldehyde (see Supporting Information File 1), which upon treatment with lithium acetylide at low temperature gave racemic propargyl alcohol **5** in 94% yield. Sonogashira coupling reaction of **5** with 4-bromoiodobenzene gave compound **6** quantitatively. The treatment of **6** with Ac_2_O and a catalytic amount of *N*,*N*-dimethyl-4-aminopyridine (DMAP) gave the acetyl ester **7** in 93% yield. The formation of 1,3-bis(4-bromophenyl)-1,3-diisopropylallene (**8**) was achieved by an S_N_2’-type reaction of **7** with iPrMgCl–CuI–LiBr at low temperature in 90% yield. This allene was easily transformed into the precursor **9** by halogen exchange with *t*-BuLi followed by the addition of C_6_F_13_I in 92% yield. Subsequently, the reaction of the diiodo precursor **9** with 2 equiv of an organozinc species derived from 4,5-bis(methylthio)TTF [19] in the presence of Pd(PPh_3_)_4_ gave racemic compound **3** in good yield (89%). In the ^13^C NMR spectrum, the allenic =C= carbon was found to be at 204 ppm, which is a typical value of the linear allene framework. In addition, the FTIR spectrum also exhibited a reasonable vibrational stretching of the C=C=C unit at 1914 cm^−1^.

Because new symmetrical allenes were subject to optical resolution, the separation of (*rac)-***3** was carried out by using a recycling HPLC method on a chiral stationary phase (DAICEL Chiralpak IA-3) with hexane/CHCl_3_/EtOH (v/v = 20:10:0.2) elution. Thus, equimolecular amounts of the optically pure allenes (+)-**3** and (−)-**3**, whose optical rotations ([α]_D_^25^) are +726 (*c* 0.853 in CH_2_Cl_2_) and −721(*c* 0.853 in CH_2_Cl_2_), respectively, were collected. Similarly, optical resolution of (*rac*)-**9** also provided (+)/(−)-**9** with optical rotations ([α]_D_^25^) of +338 (*c* 0.83 in CH_2_Cl_2_) and −341 (*c* 0.83 in CH_2_Cl_2_), respectively.

**Figure 2:**
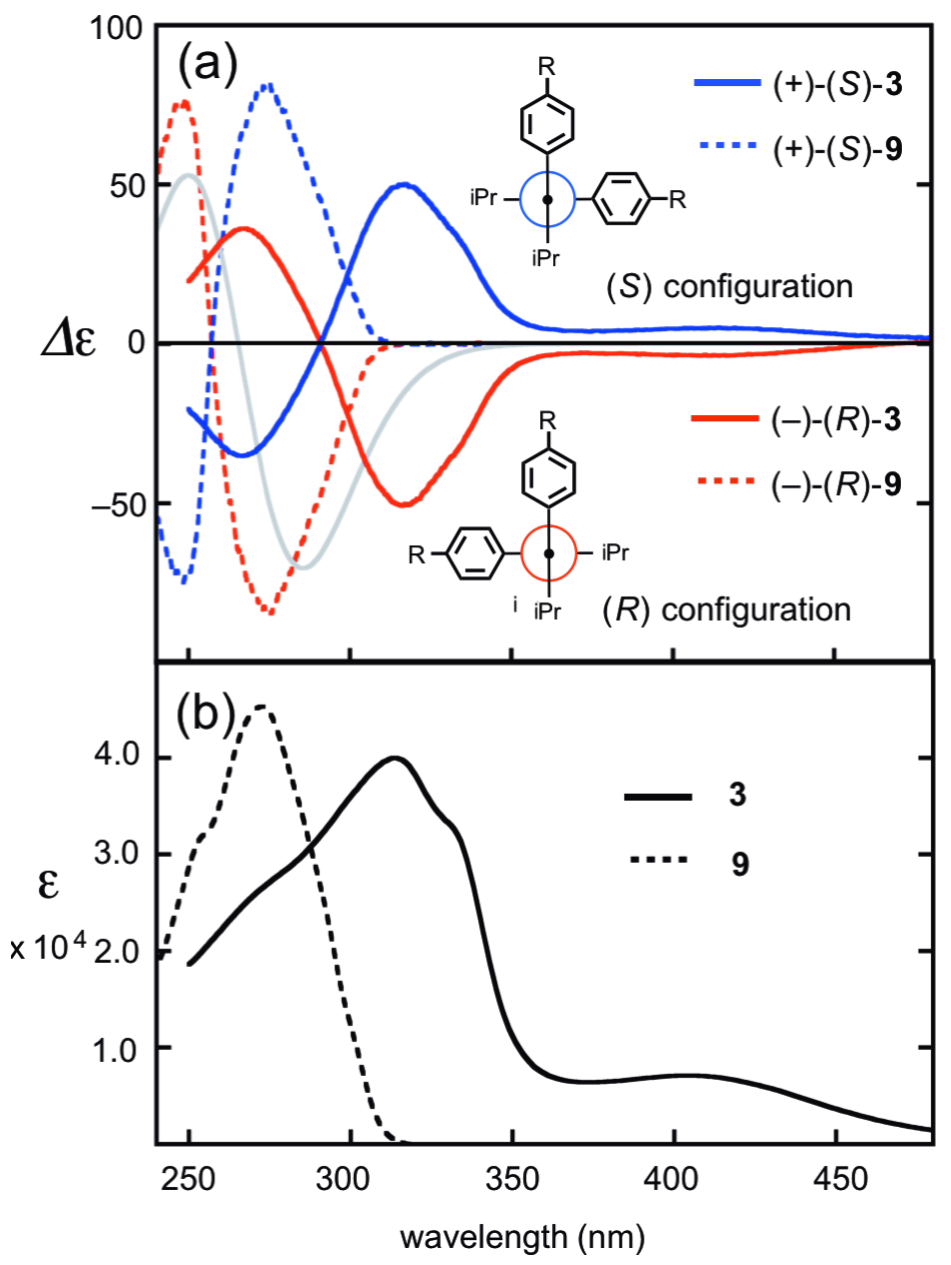
(a) ECD spectra of (*R*)-(−)-**3** (3.5 × 10^−5^ M), (*S*)-(+)-**3** (3.8 × 10^−5^ M), (*R*)-(−)-**9** (3.0 × 10^−5^ M), and (*S*)-(+)-**9** (2.9 × 10^−5^ M) in CH_2_Cl_2_ together with the simulated ECD spectra of **3** and **9** in (*R*)-configuration. (b) UV spectra of **3** (3.5 × 10^−5^ M) and **9** (3.8 × 10^−5^ M) in CH_2_Cl_2_.

Figure 2 depicted the electronic circular dichroism (ECD) spectra of the enantiomers of (+)/(−)-**3** and (+)/(−)-**9**, and their UV–vis absorption spectra. The ECD spectrum of the (−)-**9** enantiomer exhibited a clear bisignate CD curve with a negative peak at 274 nm and a positive peak at 248 nm. According to the chiral exciton coupling method [20], the spectrum trend of (−)-**9** suggests that the isomer should have the (*R*)-configuration. This assignment was also validated by spectral simulation, obtained from the electronic transition energies and the rotational strengths using TD-DFT calculations [21]. Thus, the intensive Cotton effect is associated with the exciton coupling of the two chromophores of the iodobenzene units. In the ECD spectrum of (+)-**9**, the mirror image of the trend lines of (−)-**9** was found.

The UV–vis absorption spectrum of **3** exhibits absorption maxima at 314 and 405 nm. In the ECD spectra of (+)/(−)-**3**, the Cotton effect was observed over the entire absorption range. A simulated spectrum of **3** with the (*R*)-configuration, calculated by TD-DFT (B3LYP/6-31G(d,p)), has moderate resemblance to the spectrum obtained from (−)-**3**. This result is also consistent with the hypothesis from the chiral exciton coupling method. To solidify the absolute configuration, the synthesis of (*R*)-**3** from (*R*)-**9** was carried out separately, and the ECD spectrum of the product from (*R*)-**3** was in complete accord with that of (−)-**9**. Therefore, the absolute configuration of (+)-**3** and (−)-**3** should be (+)-(*S*)-**3** and (−)-(*R*)-**3**, respectively. In the spectra, the intensive Cotton effect was observed at 270 and 316 nm as a bisignate couple, together with broad shoulder tails to ca. 500 nm. The Cotton effect associated with the HOMO–LUMO transition was relatively weak, presumably due to the long-range coupling between two TTF units in the chiral situation. Although allenes **1** and **2**, reported previously, underwent quick photoracemization, the present allenes, **3** and **9**, exhibit photochemical stability without racemization in common organic solvents. It clearly suggests that the insertion of the phenylene units effectively prevents racemization.

**Scheme 2:**
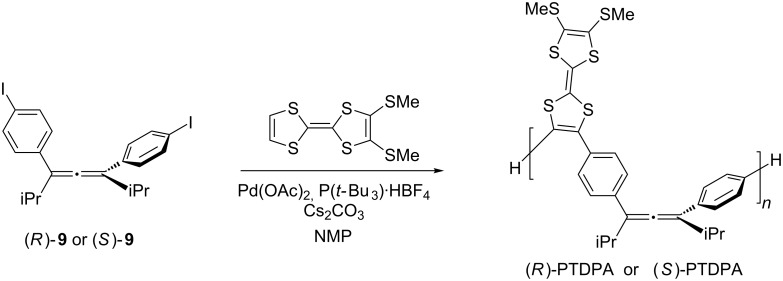
Synthesis of chiral (*R*)-PTDPA and (*S*)-PTDPA.

To examine the potential of the 1,3-diphenylallene frameworks as a stable chiral resource, a chiral alternate copolymer consisting of TTF and chiral diphenylallene (PTDPA) was prepared, and its chiroptical properties were investigated. Previously, a racemic conjugated polymer in the literature was prepared by a Suzuki–Miyaura or Yamamoto coupling reaction from (*rac*)-**8** [17]. In contrast, to the best of our knowledge, there is no example of a chiral polymer based on the 1,3-diphenylallene frameworks that can be a unit of copolymer with various types of aryl groups. At this point, we have chosen the direct arylation of TTF in the presence of a palladium catalyst as a key reaction for the chiral polymer synthesis (Scheme 2) [22]. Thus, the chiral allenes, (*R*)-**9** or (*S*)-**9,** react with an active palladium species, prepared in situ from Pd(OAc)_2_ and P(*t*-Bu)_3_·HBF_4_ salt in the presence of CsCO_3_, to give the chiral copolymer of (*R*)-PTDPA and (*S*)-PTDPA, respectively, after purification by column chromatography on polystyrene Bio-beads. By using gel permeation chromatography (GPC), the number-average molecular weights (*M*_n_) of the collected PTDPA were in the range of 2800–5600 g mol^−1^ with polydispersity index (PDI) values between 1.01–1.54. These polymers solubilized well in common organic solvents, and they were characterized by their NMR spectra.

**Figure 3:**
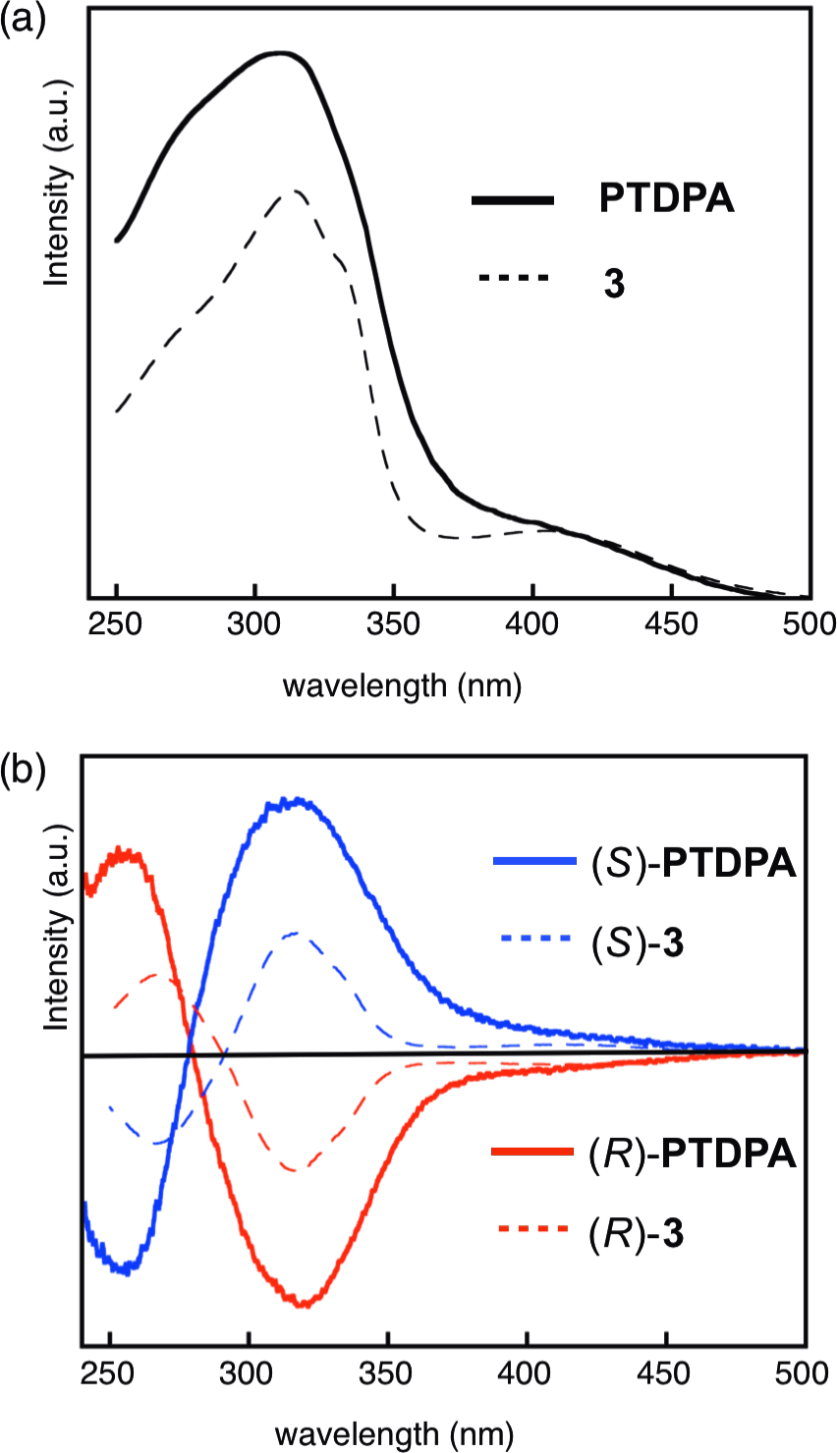
(a) UV–vis absorption spectra of **3** and PTDTA in CH_2_Cl_2_. (b) Normalized ECD spectra of (*R*)-PTDPA, (*S*)-PTDPA, (*R*)-**3**, and (*S*)-**3** in CH_2_Cl_2_.

Figure 3 depicts normalized UV–vis absorption and ECD spectra of (*R*)-PTDPA and (*S*)-PTDPA, together with (*R*)-**3** and (*S*)-**3**. The polymer exhibited absorption maxima at 310 nm and shoulder absorption at ca. 415 nm, extending up to 600 nm. The shape of the spectrum is quite similar to that of the monomer **3**. The ECD spectra of both enantiomeric polymers exhibited the mirror image. There are two intensive Cotton effects at 318 nm (λ_first_) and 254 nm (λ_second_), together with a weak ellipticity in the range of ca. 360–450 nm. The shape of the trend line of PTDPA is similar to that of **3** with the same absolute configuration at the allenic moiety except for the small blue shift of the λ_second_ value of PTDPA (254 nm). Consequently, the chiral situation of the chromophores is preserved in the polymeric structure. However, the Cotton effect associated with the TTF moieties was observed as a relatively weak band, and hence there is not any amplification owing to a higher order structure in the polymer. Moreover, the ECD intensities of both enantiomers did not change under ambient light, at least over several days. Therefore, 1,3-diphenylallene is a reliable chiral source for a chiral polymer having electron-donating units.

**Figure 4:**
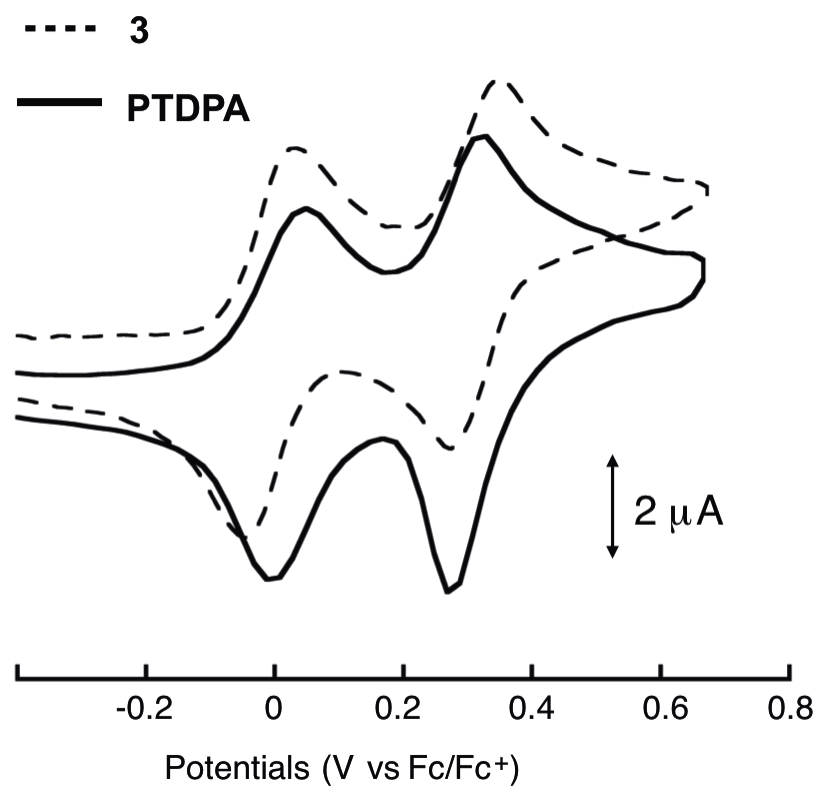
CV of **3** (7.8 × 10^−4^ M) and PTDPA in PhCN.

The electrochemical properties of **3** and PTDPA were investigated by cyclic voltammetry (CV) analyses (Figure 4 and Table 1). In CVs, there are two sets of reversible redox waves in the conventional potential range. Compound **3** exhibits two two-electron transfer waves at *E*^1^_1/2_ = −0.01 V and *E*^2^_1/2_ = 0.31 V, whereas PTDPA exhibits in a similar manner at *E*^1^_1/2_ = 0.03 V and *E*^2^_1/2_ = 0.30 V. These results suggest that the TTF units in both compounds are oxidized independently to form TTF/TTF^•+^ and, subsequently, TTF^•+^/TTF^2+^. A small positive shift of *E*^1^_1/2_ in PTDPA is presumably due to the substituent effects of the two phenylene groups in the TTF unit.

**Table 1:** Redox potentials of **3** and PTDPA.^a^

Compound	*E*^1^_1/2_	*E*^2^_1/2_

**3**	−0.01	0.31
**PTDPA**	0.03	0.30

^a^In PhCN containing 0.1 M *n*-Bu_4_ClO_4_ at 25 °C. Potentials were measured against an Ag/Ag^+^ electrode and adjusted to the Fc/Fc^+^ potential under identical conditions.

**Figure 5:**
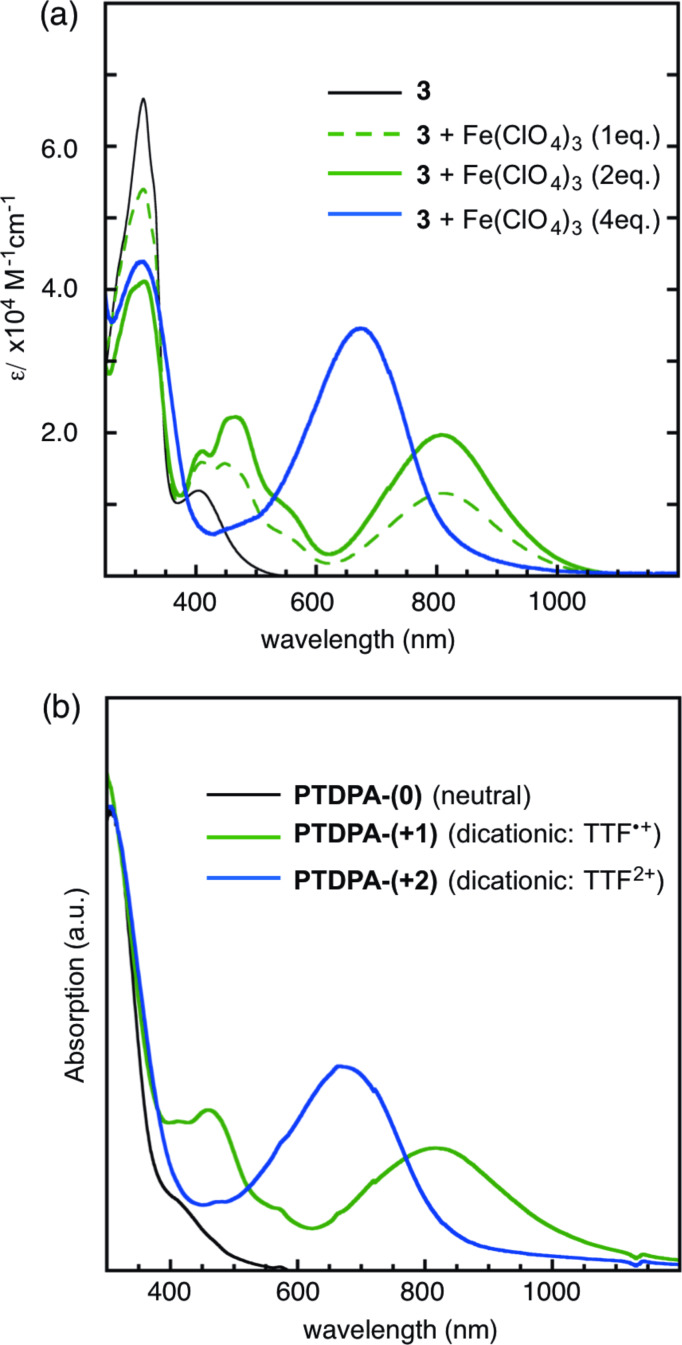
Electronic spectra of (a) **3** and its cationic species, and (b) PTDPA and its cationic states of PTDPA-(+1) and PTDPA-(+2).

To investigate the electronic structures of **3** in various redox states, cationic species of **3**^2+^ and **3**^4+^ were prepared by their reaction with an adequate amount of Fe(ClO_4_)_3_, as the oxidant, in a MeCN/CH_2_Cl_2_ (v/v = 1:4) solution (Figure 5a). During the sequential addition of between 0 to 2 equivalents of the oxidant, no isosbestic point was seen other than that of **3**/**3**^2+^. Therefore, **3**^2+^ was formed first other than **3**^•+^, suggesting that there is few intramolecular interactions between two TTF units through the central 1,3-diphenylallene moiety. The obtained spectrum of **3**^2+^ is quite similar to those of 4,5-bis(methylthio)tetrathiafulvalenylbenzene radical cation **10**^•+^, which was reported previously [7] (Table 2). In the spectrum of **3**^2+^, the absorption maximum at 810 nm is assigned to an electronic transition to the SOMO in the TTF^•+^ moieties. The value was slightly red-shifted compared with that of **10**^•+^ (775 nm). This small bathochromic shift can be ascribed to the conjugation to the central allene moiety. As for the spectrum of **3**^4+^, the absorption maximum at 667 nm, assigned to the HOMO–LUMO transition in the TTF^2+^ moieties, was also red-shifted compared with the corresponding absorption maximum (637 nm) of **10**^2+^.

**Table 2:** Absorption maxima of **3**, **10** and PTDPA.^a^

Compound	λ_max_

**3**	313 (66600), 405 (11900)
**3**^2+^	313 (41100), 412 (17500), 466 (22300), 810 (19700)
**3**^4+^	313 (43900), 667 (34600)
**10**^b^	332 (14800), 393 (3900)
**10**^•+ b^	256 (18300), 449 (11200), 775 (8100)
**10**^2+ b^	259 (21500), 646 (17900)
PTDPA-(0)	309, 410
PTDPA-(+1)	459, 816
PTDPA-(+2)	667

^a^Conditions: in CH_2_Cl_2_-MeCN (v/v = 4:1) solution at 25 °C. The molecular structure of **10** is depicted as follow:
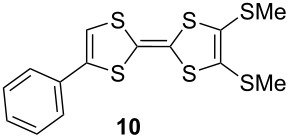
^b^Data from [7].

Similarly, cationic species of PTDPA were also produced by the sequential addition of Fe(ClO_4_)_3_ (Figure 5b). During the oxidation, three phases of PTDPA-(0), PTDPA-(+1), and PTDPA-(+2), corresponding to the oxidation stages involving TTF, TTF^•+^, and TTF^2+^, respectively, were observed during the oxidation. The spectrum of the intermediate phase of PTDPA-(+1) having absorption maxima at 459 and 816 nm are almost coincident with that of **3**^2+^ (Table 2). With excess addition of the oxidant, the third phase of PTDPA-(+2) appeared, and the spectrum was almost identical to **3**^4+^. The spectral resemblance implies that the influence of the central 1,3-diphenylallene on the electronic structure of the TTF units in PTDPA is quite small.

Finally, the ECD spectra of these cation species of **3** and PTDPA were measured (Supporting Information File 1, Figure S15). Contrary to the absorption spectra, there is no distinctive ellipticity based on the cationic species of TTF in any cationic species derived from **3** or PTDPA. Only small changes of λ_first_ and λ_second_ peaks, mainly associated with the 1,3-diphenylallene framework, were observed during the oxidation reactions. In the present chiral system, the chiroptical effects between two TTF units are small due to the long-range exciton coupling, and hence the Cotton effect derived from the electronic transition of the TTF moiety was not clearly seen. However, both chiral compounds of **3** and PTDPA did not undergo the racemization in daylight. Therefore, the chirality was also preserved in the cationic species.

## Conclusion

In this paper, we have shown the synthesis and chiroptical properties of a novel chiral TTF dimer linked by a 1,3-diphenylallene framework for the purpose of the development of a stable chiral source without racemization under ambient conditions. The title compound was synthesized from 1,3-bis(4-iodophenyl)-1,3-diisopropylallene (**9**) and organozinc species derived from 4,5-bis(methylthio)TTF. Optical resolution of the enantiomer **3** and its precursor **9** was achieved by a recyclable HPLC on a chiral stationary phase. The ECD spectra were measured, and the obtained spectra allowed for the validation of the absolute configurations based on the TD-DFT method. Although the observed Cotton effect associated with TTF moieties is relatively weak, each chiral allene is stable towards the racemization under ambient light. A chiral copolymer based on TTF and a chiral 1,3-diphenylallene as the main chain scaffold (PTDPA) was also prepared using a direct C–H activation of the TTF framework. The resultant chiral polymers exhibited the characteristic Cotton effect associated with the central chiral 1,3-diphenylallene moieties. The polymer also did not undergo racemization under daylight. Moreover, electrochemical properties were also investigated by the CV method. Cationic species were prepared by the addition of Fe(ClO_4_)_3_ as the oxidant, and their UV and ECD spectra were also recorded. Other molecular designs, including a copolymer based on the 1,3-diphenylallene units, are currently underway to create valuable chiral molecules.

## Experimental

4,5-Bis(methylthio)TTF was prepared according to literature procedures [19]. Dehydrated THF and Et_2_O were purchased from Wako Pure Chemical Industries, LTd. (Super Dehydrated, Stabilizer Free, H_2_O < 10ppm). Anhydrous ZnCl_2_ (99%) was purchased from Wako Pure Chemical Industries, and dried in vacuo for 4 h with heating over 220 °C. Other commercially available materials were used as received. Column chromatography was carried out using Kanto chemical silica gel 60N, 60–210 μm meshes. ^1^H and ^13^C NMR spectra were recorded on Bruker AVANCE-III-400 (400 MHz for ^1^H, 100 MHz for ^13^C) or on Bruker AVANCE-III-600 (600 MHz for ^1^H, 150 MHz for ^13^C). Spectra are reported (in δ) referenced to internal Me_4_Si. Mass spectra were recorded on Thermo Scientific, Exactive Plus Orbitrap Mass Spectrometer with atmospheric pressure chemical ionization (APCI) probe. IR spectra were recorded on JASCO FT/IR-610 spectrometer. Melting points were determined with Yanaco melting point apparatus. Elemental analyses were performed on Perkin Elmer PE 2400-II CHNA/O analyzer. Optical resolution was carried out with recyclable preparative HPLC (JAI Model LC-9204) equipped with Daicel CHIRALPAK-IA3 column (20 Ø × 250 mm). The optical rotations were measured with JASCO P-1300 spectropolarimeter in 1 dm quartz cell at 24 °C. Electronic circular dichroism (ECD) spectra were recorded on JASCO J-725 spectrodichrometer. The spectra were combined after the baseline correction of each measurement. cyclic voltammetry (CV) measurements were performed on Hokuto Denko HZ-5000 electrochemical analyzer. GPC analysis for the polymer products was carried out at 40 °C on a Shodex GPC apparatus equipped with two SB-806M HQ GPC columns (Showa Denko K. K.) and a UV detector. DMF was used as the eluent at a flow rate of 0.5 mL/min. Polystyrene standards with a narrow distribution of molecular weight (*M*_w_: 580–377, 400) were used for molecular weight calibration.

**Synthesis of 8:** To a solid mixture of dried CuI (5.7 g, 30 mmol) and LiBr (2.6 g, 30 mmol) was added dropwise acetyl ester **7** (6.7 g, 15 mmol) in THF (170 mL) during 15 min at rt, and the mixture was cooled to −78 °C. Then, freshly prepared iPrMgCl (30 mmol) in THF (15 mL) was added dropwise into the mixture at −78 °C. After stirring for 1 h at −78 °C, the mixture was further stirred for 14 h at rt. The resultant mixture was poured into saturated aqueous NH_4_Cl solution, and the products were extracted by Et_2_O. The organic phase was washed with brine and dried over MgSO_4_. After evaporation, the crude products were purified on silica gel column chromatography with the elution of hexane. Removal of the solvent afforded **8** as colourless oil (5.8 g, 90%): MS (GC) *m/z* = 432 (50%, M^+^: C_21_H_22_^79^Br_2_), 434 (100%, M^+^: C_21_H_22_^79^Br^81^Br), and 436 (50%, M^+^: C_21_H_22_^81^Br_2_); ^1^H NMR (400 MHz, CDCl_3_) δ 7.42 (d, *J* = 8.6 Hz, 2H), 7.27 (d, *J* = 8.6 Hz, 2H), 2.91 (septet, *J* = 6.8 Hz), 1.17 (d, *J* = 6.8 Hz, 3H), 1.15 (d, *J* = 6.8 Hz, 3H); ^13^C NMR (100 MHz, CDCl_3_) δ 203.0, 135.6, 131.7, 128.0, 120.7, 117.1, 28.8, 22.7, 22.4; IR (neat): 2961, 2926, 2870, 1919, 1489, 1464, 1074, 1009, 825 cm^−1^; HRMS (APCI-orbitrap) calcd for C_21_H_22_Br_2_ (M^+^) 432.0088; found, 432.0089.

**Synthesis of 9:** To a solution of 1,3-bis(4-bromophenyl)allene derivative **8** (1.0 g, 2.3 mmol) in Et_2_O (50 mL) was added dropwise *t*-C_4_H_9_Li (6.0 mL, 9.8 mmol) at −78 °C under Ar atmosphere. After stirring for 30 min at the same temperature, C_6_F_13_I (1.3 mL, 5.84 mmol) was added dropwise by syringe. The resultant mixture was stirred for 3 h at −78 °C, and then saturated aqueous NH_4_Cl solution was poured into the solution at −78 °C. After allowing to warming up to rt, the mixture was extracted by Et_2_O. The organic phase was washed with brine and dried over Na_2_SO_4_. After removal of the solvent, the crude product was purified by column chromatography on silica gel with hexane elution to give colourless oil of **9** (1.0 g, 92%): MS (APCI) *m/z* = 528 (M^+^); ^1^H NMR (400 MHz, CDCl_3_) δ 7.62 (d, *J* = 8.6 Hz, 2H), 7.14 (d, *J* = 8.6 Hz, 2H), 2.94 (septet, *J* = 6.7 Hz, 1H), 1.17 (d, *J* = 6.8 Hz, 2H), 1.15 (d, *J* = 6.8 Hz, 2H); ^13^C NMR (100 MHz, CDCl_3_) δ 202.9, 137.6, 136.0, 128.1, 117.0, 92.2, 28.6, 22.7, 22.4; IR (neat): 2961, 2926, 2869, 1918, 1483, 1463, 1383, 1216 cm^−1^; HRMS (APCI-orbitrap) calcd for C_21_H_22_I_2_ (M^+^) 527.98108; found, 527.98108.

**Synthesis of 3:** To a solution of 4,5-bis(methylthio)TTF (640 mg, 2.2 mmol) [20] in THF (30 mL) was added dropwise *n*C_4_H_9_Li (1.3 mL, 2.2 mmol) at –78 °C under Ar atmosphere. After the mixture was stirred for 90 min, a suspension of ZnCl_2_ (377 mg, 2.8 mmol) in THF (3 mL) was added at −65 °C. The solution was further stirred for 30 min, then 1,3-bis(4-iodophenyl)allene derivative **9** (458 mg, 0.87 mmol) in THF (2.5 mL) and Pd(PPh_3_)_4_ (100 mg, 0.087 mmol) were added into the mixture at –20 °C. The resultant mixture was stirred for 14 h at rt, and then poured into saturated aqueous NH_4_Cl solution. The product was extracted by Et_2_O, and the organic phase was washed with saturated brine and dried over MgSO_4_. The crude product was purified by column chromatography on silica gel with CH_2_Cl_2_/hexane (v/v = 1:4). Recrystallization from CH_2_Cl_2_/MeOH (v/v = 1:1) solution gave **3** (664 mg, 89%): orange powder; mp: 77.8–79.2 °C; MS (APCI) *m/z* = 865 (M^+^ + H); ^1^H NMR (400 MHz, CDCl_3_) δ 7.40 (d, *J* = 8.6 Hz, 4H), 7.34 (d, *J* = 8.6 Hz, 4H), 6.48 (s, 2H), 2.97 (sept, *J* = 6.8 Hz, 2H), 2.44 (s, 6H), 2.43 (s, 6H), 1.19 (t, *J* = 6.8 Hz, 12H); ^13^C NMR (100 MHz, CDCl_3_) δ 204.2, 136.9, 136.0, 130.8, 127.8, 127.7, 126.7, 126.6, 117.4, 114.8, 112.8, 107.3, 28.7, 22.8, 22.5, 19.4; IR (KBr): 2958, 2918, 2866, 1914, 1568, 1499, 1418, 835, 759 cm^−1^; anal. calcd for C_37_H_36_S_12_: C, 51.35; H, 4.19; found: C, 51.36; H, 4.25.

**Synthesis of (*R*)/(*S*)-PTDPA:** A mixture of Pd(OAc)_2_ (8.9 mg, 40 μmol), P(*t*-Bu)_3_·HBF_4_ (35 mg, 120 mmol), Cs_2_CO_3_ (290 mg, 2.8 mmol) in degassed NMP (1 mL) was stirred for 20 min at 100 °C under Ar atmosphere. Then, (*R*)-**3** (80 mg, 151 μmol) and 4,5-bis(methylthio)TTF (67 mg, 226 μmol) were added to the solution, and the reaction mixture was stirred for 3 days at 100 °C. After cooling to rt, the solvent was removed under reduced pressure, and then the residue and *N*,*N*-diethylphenylazothioformamide (100 mg, 40 μmol), as a metal scavenger, were dispersed in THF (2 mL). The resultant suspension was stirred for 30 min at rt, then further for 1 h at 100 °C. After cooling at rt, the solution was poured into CH_3_OH (150 mL). The resulted precipitates were collected by filtration, washing with CH_3_OH. The residue on the filter was collected, and then purified by column chromatography on non-polar polystyrene gel (Bio-beads S-X3 Support) with the elution of toluene. The crude product was further purified reprecipitation with toluene-hexane, yielding copolymer of (*R*)-PTDPA as an orange-brown powder (24 mg, 26%). The number-average molecular weight (*M*_n_) was estimated to be 5.6 × 10^3^, and its distribution (*M*_n_/*M*_w_) was estimated to be 1.01: orange-brown powder; ^1^H NMR (400 MHz, CDCl_3_) δ 7.13–7.36, 6.46, 2.89, 2.40, 1.14. In similar manner, (*S*)-PTDPA was obtained from the reaction of (*S*)-**3** in 31% yield. Its *M*_n_ value was estimated to be 2.8 × 10^3^ and its distribution was estimated to be 1.54.

## Supporting Information

File 1 Experimental procedures, characterization data, copies of ^1^H and ^13^C NMR charts, recyclable chiral HPLC chart and DFT calculation summary.
