# Supplementary material for: Chiroptical properties of 1,3-diphenylallene-anchored tetrathiafulvalene and its polymer synthesis
Source: Beilstein J Org Chem. 2015 Jun 8;11:972–9. doi: 10.3762/bjoc.11.109 (PMC4505084; doi:10.3762/bjoc.11.109)
Supplement: File 1 — Experimental procedures, characterization data, copies of 1H and 13C NMR charts, recyclable chiral HPLC chart and DFT calculation summary. [file Beilstein_J_Org_Chem-11-972-s001.pdf]

**Supporting Information**
**for**

# Chiroptical properties of 1,3-diphenylallene-anchored tetrathiafulvalene and its polymer synthesis

Masashi Hasegawa*[1], Junta Endo[1], Seiya Iwata[1], Toshiaki Shimasaki[2], and Yasuhiro Mazaki*[1]

Address: [1]Department of Chemistry, School of Science, Kitasato University, 1-15-1 Kitasato, Minami-ku, Sagamihara, Kanagawa 252-0373, Japan and [2]Graduate School of Engineering, Chiba Institute of Technology, 2-17-1 Tsudanuma, Narashino, Chiba 275-0016, Japan

Email: Masashi Hasegawa - masasi.h@kitastato-u.ac.jp; Yasuhiro Mazaki - mazaki@kitasato-u.ac.jp

*Corresponding author

## Experimental procedures, characterization data, copies of $^{1}$H and $^{13}$C NMR charts, recyclable chiral HPLC chart and DFT calculation summary

**Contents**

S1. Synthesis of **4** p. S2
S2. Synthesis of **7** from **4** p. S3
S3. Figure S1. $^{1}$H and $^{13}$C NMR Chart of **5** p. S6
S4. Figure S2. $^{1}$H and $^{13}$C NMR Chart of **6** p. S7
S5. Figure S3. $^{1}$H and $^{13}$C NMR Chart of **7** p. S8
S6. Figure S4. $^{1}$H and $^{13}$C NMR Chart of **8** p. S9
S7. Figure S5. $^{1}$H and $^{13}$C NMR Chart of **9** p. S10
S8. Figure S6. $^{1}$H and $^{13}$C NMR Chart of **3** p. S11
S9. Figure S7. $^{1}$H NMR Chart of (a) (*R*)-PTDPA and (b) (*S*)- PTDPA p. S12
S10. Figure S8. Chiral HPLC Chart of (a) **9** and (b) **3** p. S13
S11. DFT Calculations of **9** and **3** p. S14
S12. TD-DFT Calculations and MO diagram of **9** and **3** p. S22
S13. ECD Spectra of Cationic Species of **9** and **3** p. S24
S14. Examination of the Photoracemization p. S24
S15. References p. S25

## S1. Synthesis of 4

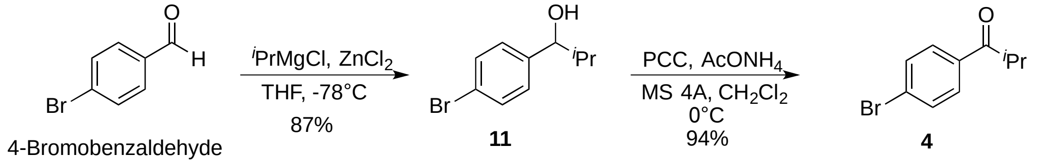


**Scheme S1.** Synthesis of **4**.

Although 1-(4-bromophenyl)-2-methylpropanone (**4**) is know compound, we modified its synthesis in order to obtain in a large quantity. Scheme 1 is depicted the synthesis of **4** via alcohol **11**.[s1]

1. Synthesis of **11**

To a suspension of $ZnCl_2$ (9.4 g, 69 mmol), which was dried for 4h at 200 °C in vacuo, in THF (100 mL), $^{i}$PrMgCl (206 mmol) in THF (100 mL) was added dropwise under Ar atmosphere at rt. The resultant colorless suspension was stirred for 1h, and then cooled at 0°C. 4-Bromobenzaldehyde (12.7 g, 69 mmol) in THF (69 mL) was added and stirred for 14h at 0°C. Several portions of HCl (2M) were added into the reaction mixture at 0°C, and then the products were extracted by $Et_2O$ three times. The organic layer was washed with saturated brine and dried over $Na_2SO_4$. The volatiles were removed under reduced pressure, and the resultant crude products were purified on silica gel column chromatography with $CH_2Cl_2$ as the eluent to give pale yellow oil of **11** (13.6 g, 87%). Data for **10**: MS (GC) *m/z* = 228 (100%, $M^+$: $C_{10}H_{13}Br^{79}O$), 230 (100%, $M^+$: $C_{10}H_{13}Br^{81}O$). $^1$H NMR (600 MHz, $CDCl_3$) δ 7.43 (2H, d, *J* = 8.4 Hz), 7.14 (2H, d, *J* = 8.4 Hz), 4.28 (1H, dd, *J* = 6.6 and 1.2 Hz), 2.24 (1H, d, *J* = 1.2 Hz), 1.83-1.91 (1H, m), 0.94 (3H, d, J = 6.6 Hz), 0.77 (3H, d, J = 6.6 Hz). $^{13}$C NMR (150 MHz, $CDCl_3$) δ 142.7, 13.14, 128.4, 121.3, 79.2, 35.2, 19.0, 18.1.

2. Synthesis of **4**

To a suspension of **11** (13.6 g, 60 mmol), $AcONH_4$ (13.8 g, 178 mmol), and molecular sieves 4Å (30 g) in $CH_2Cl_2$ (500 mL), PCC (19.2 g, 89 mmol) was added portionwise at 0°C. The mixture was allowed to warming up to rt, and stirred for 14h. The mixture was filtrated through a pad of celite with $CH_2Cl_2$. The volatiles were removed under reduced pressure, and the products were purified on silica gel column chromatography with

$CH_2Cl_2$ as the eluent to give pale yellow oil of **4** (12.7 g, 94%). Data for **4**: MS (GC) *m/z* = 226 (100%, $M^+$: $C_{10}H_{11}Br^{79}O$), 228 (100%, $M^+$: $C_{10}H_{11}Br^{81}O$). $^1$H NMR (400 MHz, $CDCl_3$) δ 7.81 (2H, d, $J$ = 8.6 Hz), 7.59 (2H, d, $J$ = 8.6 Hz), 3.49 (1H, sept, $J$ = 6.8 Hz), 1.2 (6H, d, $J$ = 6.8 Hz). $^{13}$C NMR (100MHz, $CDCl_3$) δ 203.3, 135.0, 142.7, 132.0, 129.9, 127.9, 35.5, 19.1.

## S2. Synthesis of 7 from 4

1. Synthesis of (*rac*)-**5**

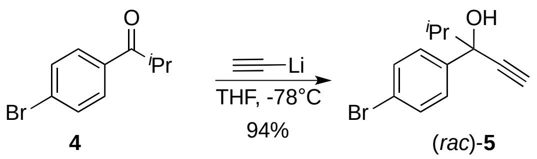


4 (*rac*)-**5**

**Scheme S2.** Synthesis of **5**.

In 1L three-necked flask containing THF (120 mL) under Ar atmosphere, acetylene gas, purified through cold trap and sulfuric acid in the upper course, was filled from a needle as the gas-inlet at -78°C. $^n C_4H_9Li$ (in hexane, 27.4 mL, 45 mmol) was carefully added dropwise to the solution. The mixture was stirred for 30min, and the inlet gas was stopped. Then, compound **4** (6.8 g, 30 mmol) in THF (50 mL) was added dropwise at -78°C. The mixture was slowly allowed to warming to rt, and stirred for 16h at rt. The reaction was quenched by the addition of saturated aqueous $NH_4Cl$ solution, and the mixture was extracted by $Et_2O$ three times. The organic phase was washed with saturated brine and dried over $MgSO_4$. After removal of the volatiles, the residue was purified by column chromatography on silica gel with $CH_2Cl_2$ as the eluent to give pale yellow powder of **5** (7.1 g, 94%). Data for **5**: Mp. 39.8-40.0 °C. MS (GC) *m/z* = 252 (100%, $M^+$: $C_{12}H_{13}Br^{79}O$) and 254 (100%, $M^+$: $C_{12}H_{13}Br^{81}O$). $^1$H NMR (600 MHz, $CDCl_3$) δ 7.46-7.49 (4H, m), 2.69 (1H, s), 2.37 (1H, s), 2.06 (1H, sept, 6.6 Hz), 1.05 (3H, d, $J$ = 6.6 Hz), 0.83 (3H, d, $J$ = 6.6 Hz). $^{13}$C NMR (150 MHz, $CDCl_3$) δ 142.5, 131.0, 128.0, 121.8, 84.6, 76.6, 75.2, 40.2, 17.7, 17.2. IR (KBr) 3366, 3291, 2993, 2971, 2925, 2873, 2113, 1914, 1651, 1586, 1489, 1469, 1397, 1386, 1346, 1306 $cm^{-1}$. Anal. Calcd for $C_{12}H_{13}BrO$: C, 56.94 %; H, 5.18 % Found: C, 57.21%; H, 5.17 %.

2. Synthesis of (*rac*)-**6**

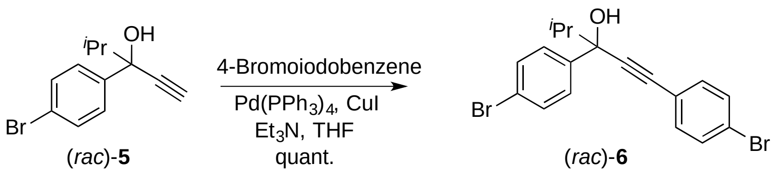


**Scheme S3.** Synthesis of **6**.

A mixture of **5** (12.7g, 50 mmol), 4-bromoiodobenzene (17.0 g, 60 mmol), CuI (0.95 g, 5.0 mmol), $Et_3N$ (5 mL), and $Pd(PPh_3)_4$ (1.0 g, 0.88 mmol) in THF (220 mL) was stirred for 16h under Ar atmosphere. Then, the resultant mixture was filtrated through a pad of celite with $CH_2Cl_2$. After removal the volatiles, the residue was purified by column chromatography on silica gel with $CH_2Cl_2$ as the eluent to give yellow oil of **6** (20.3 g, 100 %). Data for **6**: MS (APCI) *m/z* = 406 (19%, $M^+$), 389 (100%, $M^+$-OH). $^1$H NMR ($CDCl_3$, 600 MHz) δ 7.48-7.50 (2H, m), 7.43-7.47 (4H, m), 7.30-7.32 (2H, m), 2.60 (1H, s), 2.11 (1H, sept, *J* = 6.6 Hz), 1.07 (3H, d, *J* = 6.6 Hz), 0.87 (3H, d, *J* = 6.6 Hz). $^{13}$C NMR ($CDCl_3$, 150 MHz) δ 142.8, 133.2, 131.7, 131.1, 128.0, 123.0, 121.8, 121.4, 91.2, 86.0, 77.1, 40.5, 18.0, 17.5. IR (KBr) 3434, 2965, 2224, 1901, 1782, 1731, 1645 cm$^{-1}$. HRMS (APCI-orbitrap) calcd. for $C_{18}H_{16}Br_2O$ ($M^+$) 405.9568, found 405.9544.

3. Synthesis of (*rac*)-**7**

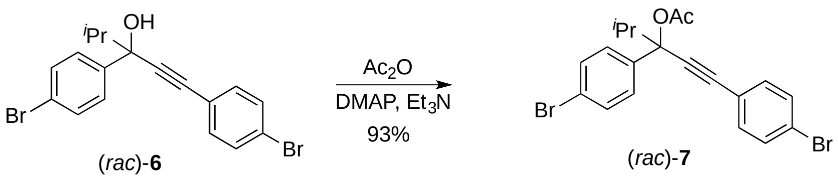


**Scheme S4.** Synthesis of **7**.

To a solution of **6** (5.0 g, 12 mmol), DMAP (0.23 g, 1.9 mmol) in $CH_2Cl_2$ (30 mL), $Et_3N$ (30 mL, excess) and $Ac_2O$ (5.8 mL, 62 mmol) were added under Ar atmosphere at rt. The mixture was stirred for 16h, and then saturated aqueous $NH_4Cl$ was added. The product was extracted by $Et_2O$, and the combined organic phase was washed with saturated brine and dried over $MgSO_4$. The volatiles were removed in vacuo to give colorless solid of **7** (5.2 g, 93 %). Compound **7** is unstable to silica gel, and therefore further purification was not carried out. Data for **7**: Mp. = 84.0-86.5 °C. MS (APCI) *m/z* = 399 (50%, $M^+$-OAc: $C_{18}H_{15}Br^{79}{}_2$), 391 (100%, $M^+$-OAc: $C_{18}H_{15}Br^{79}Br^{81}$), 393 (50%,

$M^+$-OAc: $C_{18}H_{15}Br^{81}{}_2$), 408 (0.2%, $M^+$: $C_{20}H_{18}Br^{79}{}_2O_2$), 410 (0.4%, $M^+$: $C_{20}H_{18}Br^{79}Br^{81}O_2$), 412 (0.2%, $M^+$: $C_{20}H_{18}Br^{81}{}_2O_2$). $^1$H NMR ($CDCl_3$, 600 MHz) δ 7.34-7.39 (4H, m), 7.30-7.34 (4H, m), 2.18 (1H, sept, $J$ = 6.6 Hz), 1.98 (3H, s), 1.14 (3H, d, $J$ = 6.6 Hz), 0.68 (3H, d, $J$ = 6.6 Hz). $^{13}$C NMR ($CDCl_3$, 150 MHz) δ 169.4, 140.1, 133.5, 131.6, 131.3, 127.6, 123.1, 121.8, 121.3, 88.0, 86.3, 82.6, 40.3, 21.7, 18.1, 17.2. IR (KBr) 3066, 2969, 2932, 2874, 2229, 1902, 1748, 1489 cm$^{-1}$. Anal. Calcd for $C_{20}H_{18}Br_2O_2$: C, 53.36 %; H,4.03 % Found: C, 53.32 %; H, 4.03 %.

## S3. Figure S1. $^{1}$H and $^{13}$C NMR Chart of 5

(a) $^{1}$H NMR (600 MHz, $CDCl_3$)

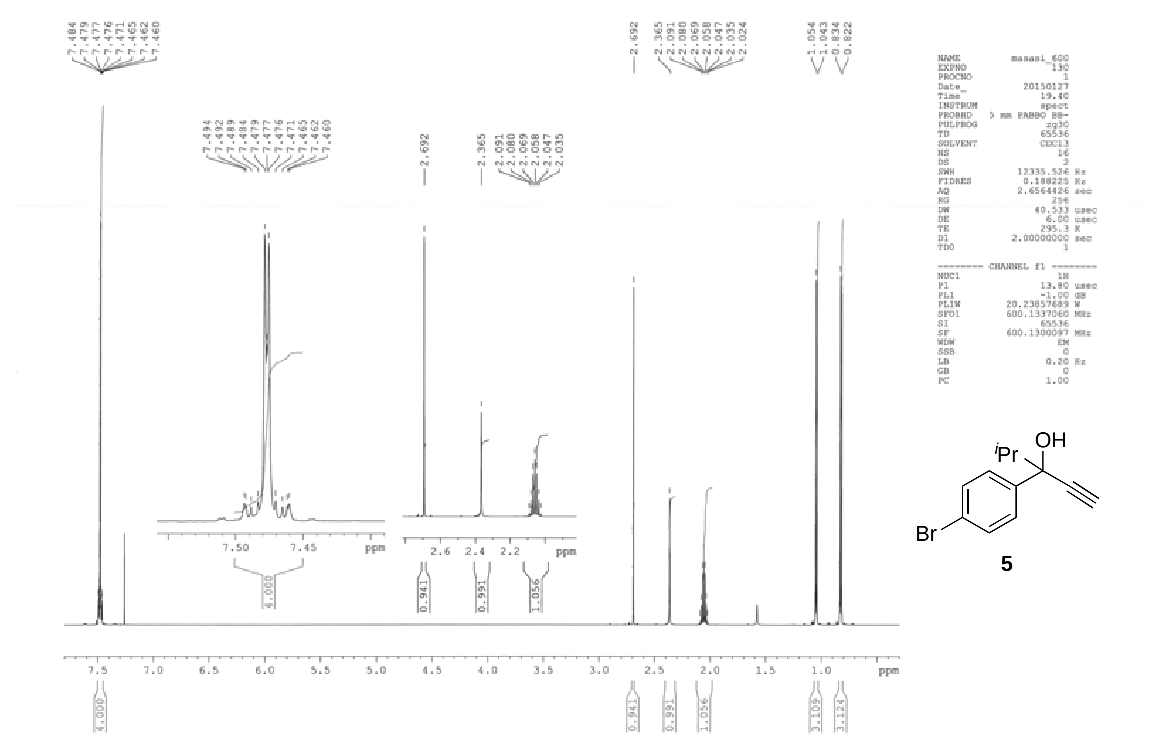


(b) $^{13}$C NMR (150 MHz, $CDCl_3$)

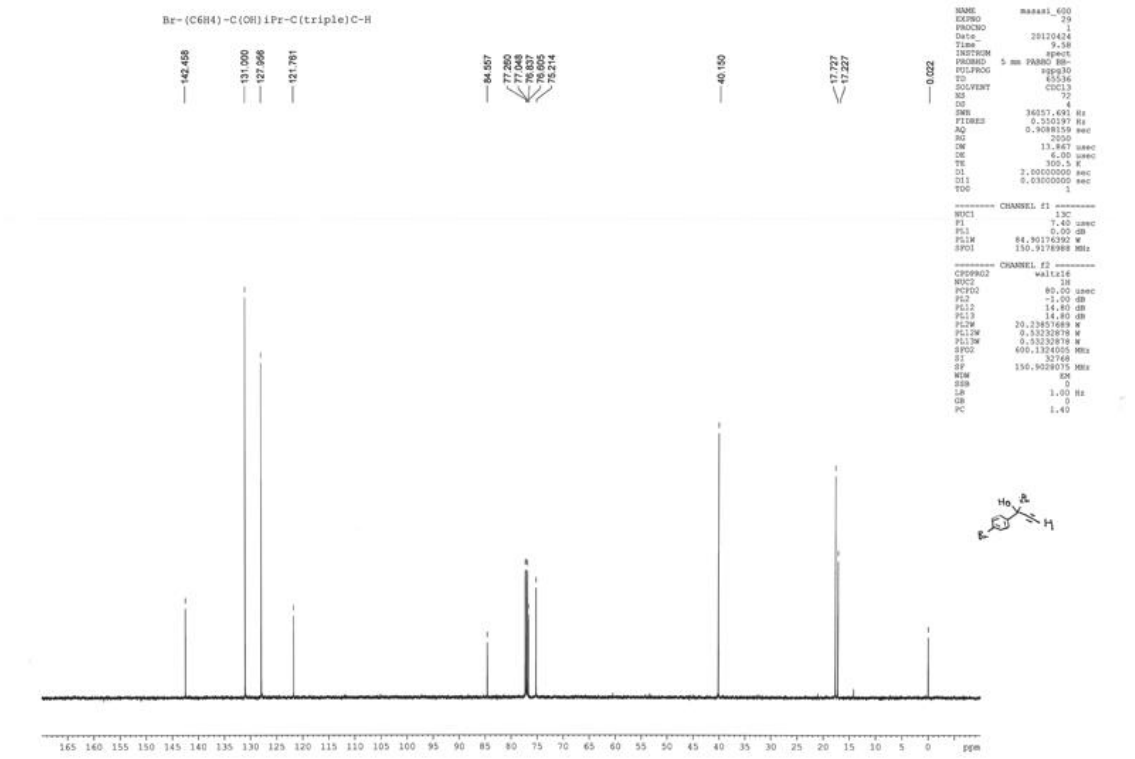

## S4. Figure S2. $^{1}$H and $^{13}$C NMR Chart of 6

(a) $^{1}$H NMR (600 MHz, $CDCl_3$)

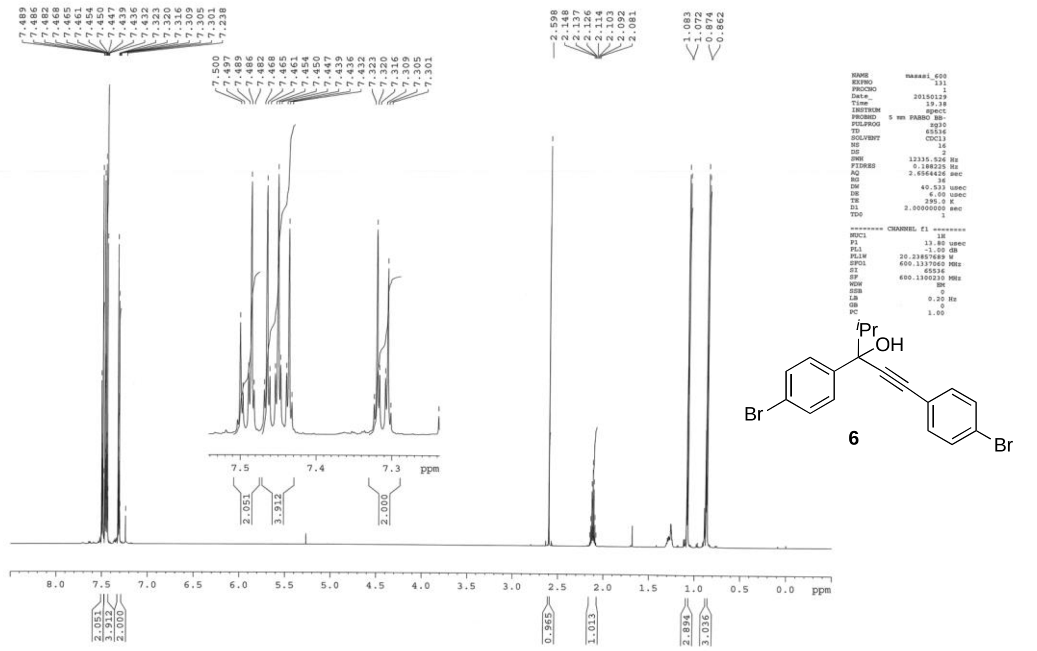


(b) $^{13}$C NMR (150 MHz, $CDCl_3$)

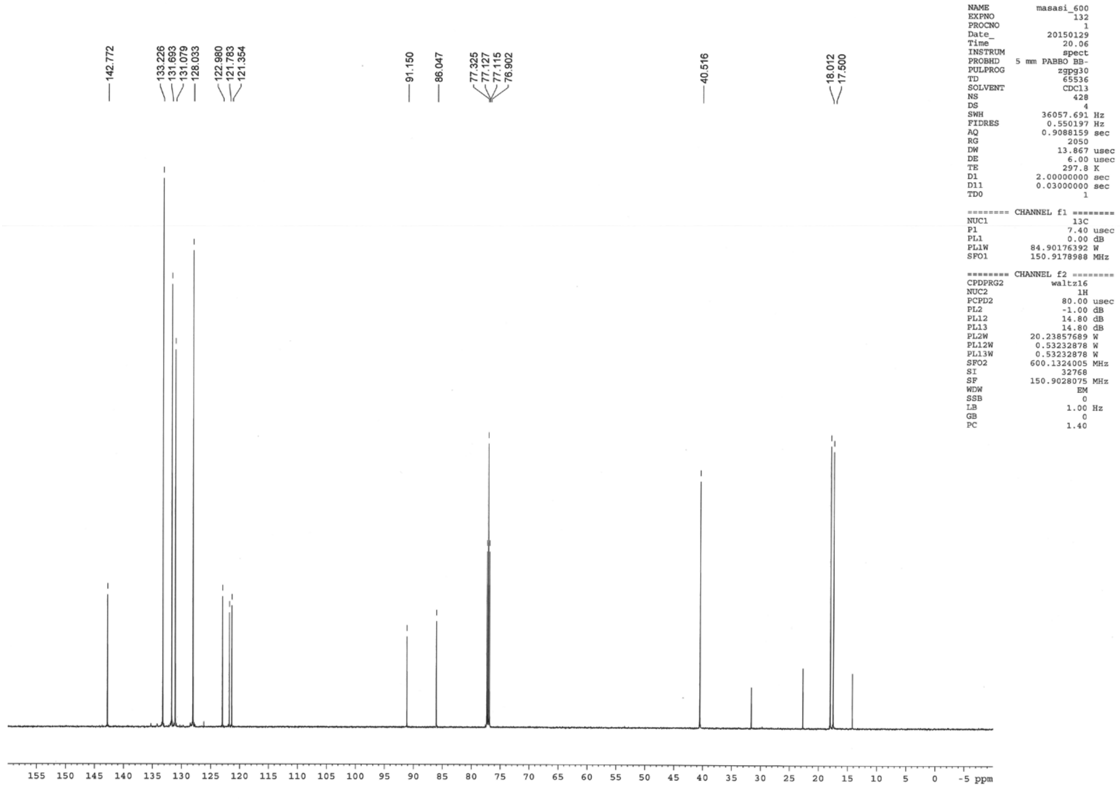

**S5. Figure S3. $^{1}$H and $^{13}$C NMR Chart of 7**

(a) $^{1}$H NMR (600 MHz, $CDCl_3$)

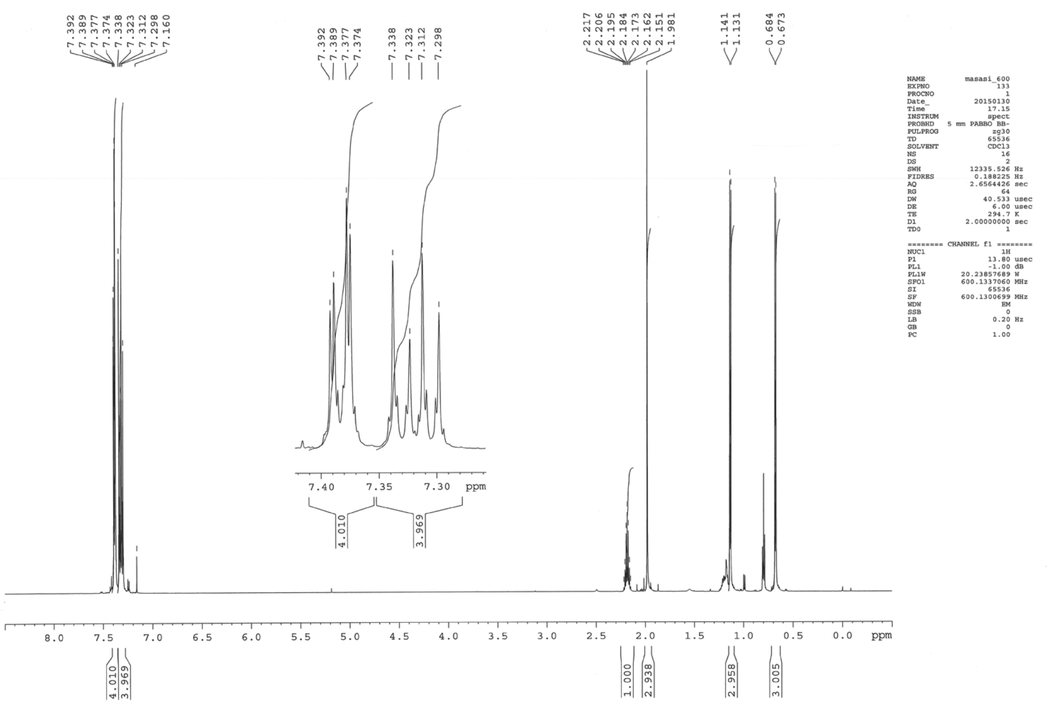

(b) $^{13}$C NMR (150 MHz, $CDCl_3$)

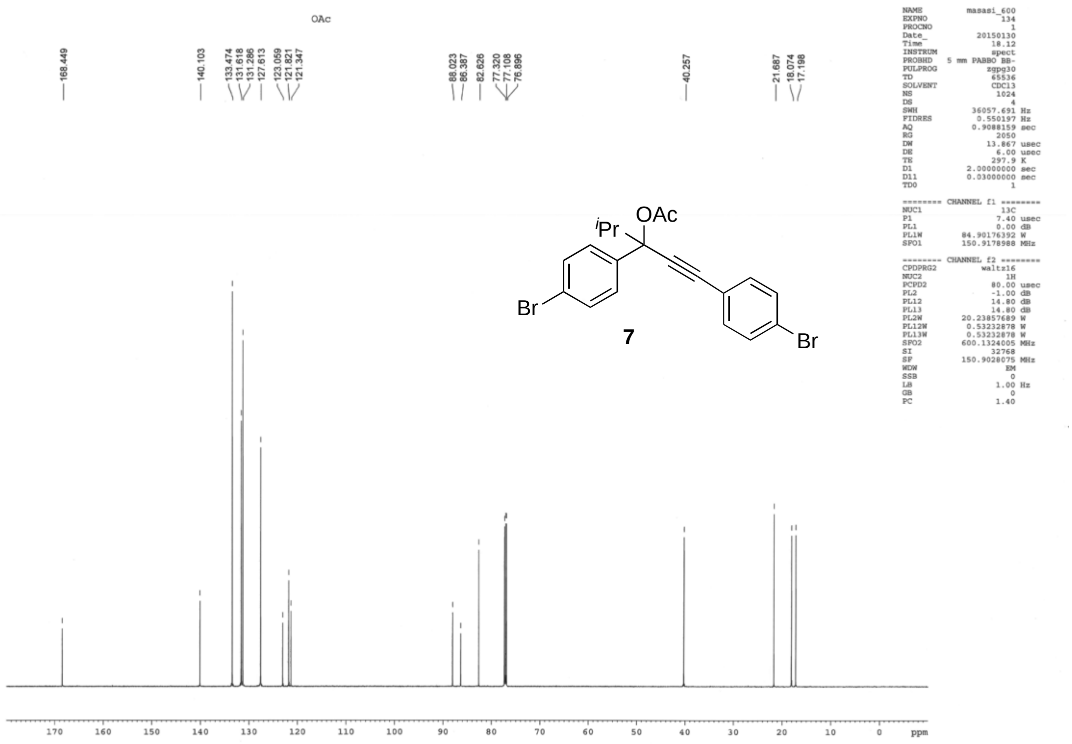

## S6. Figure S4. $^1$H and $^{13}$C NMR Chart of 8

(a) $^1$H NMR (400 MHz, $CDCl_3$)

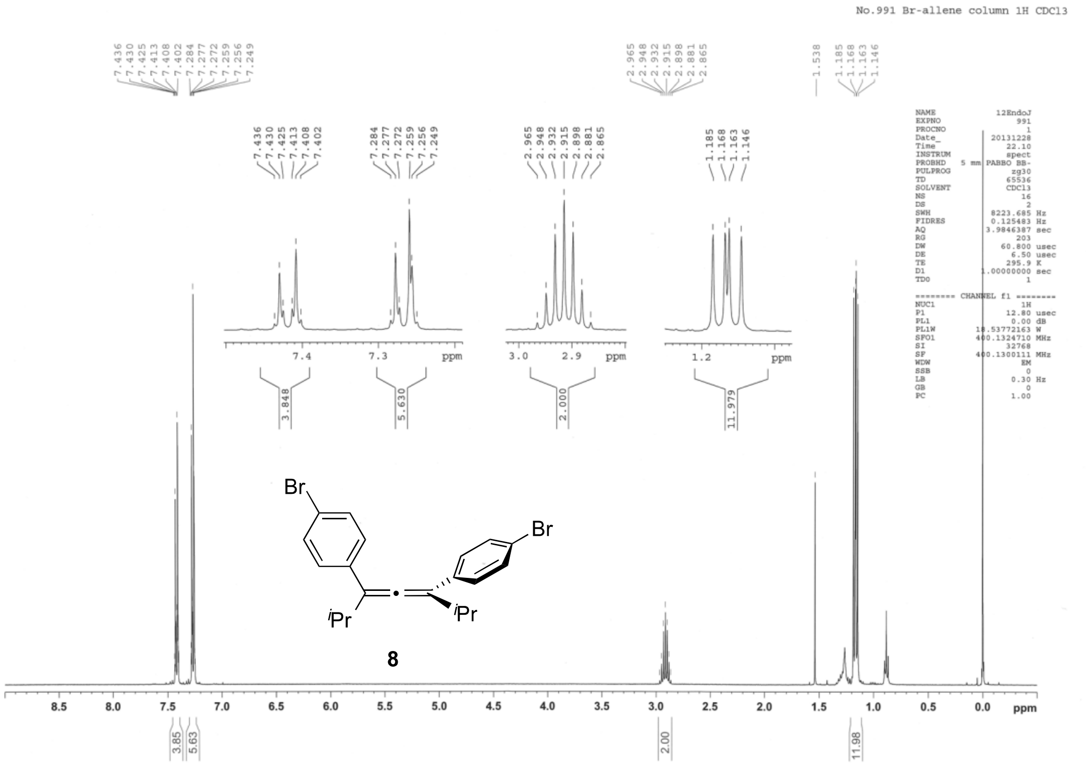


(b) $^{13}$C NMR ($CDCl_3$, 100 MHz)

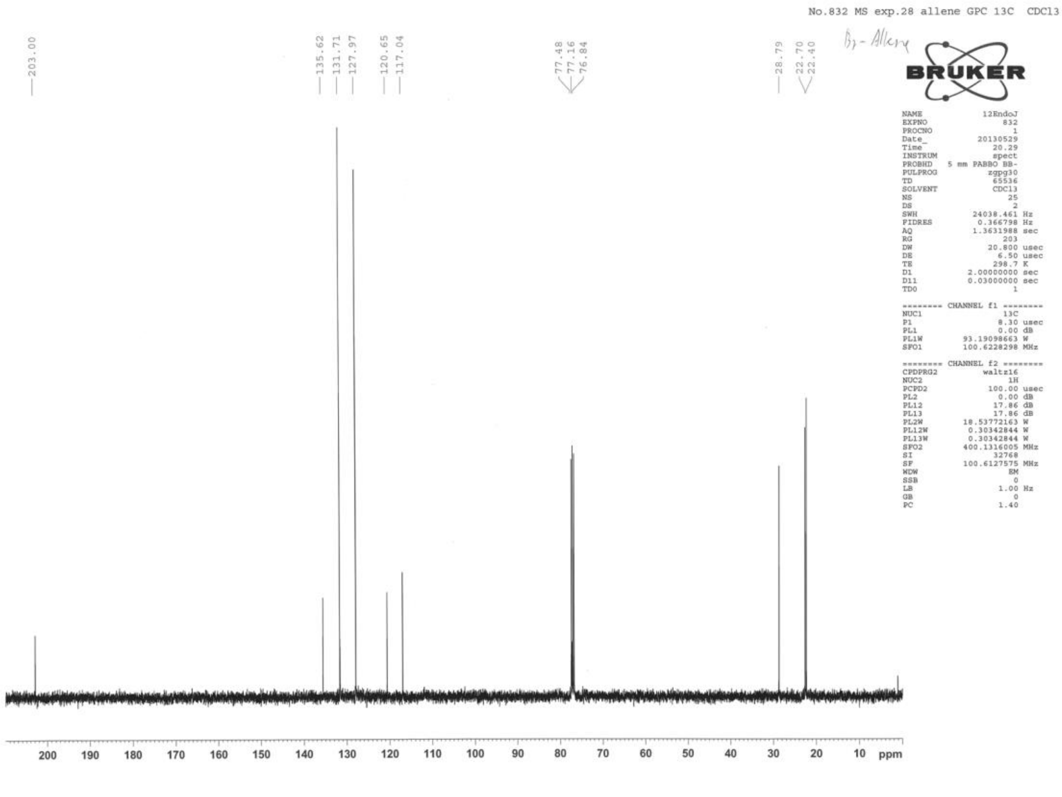

## S7. Figure S5. $^{1}$H and $^{13}$C NMR Chart of 9

(a) $^{1}$H NMR (400 MHz, $CDCl_3$)

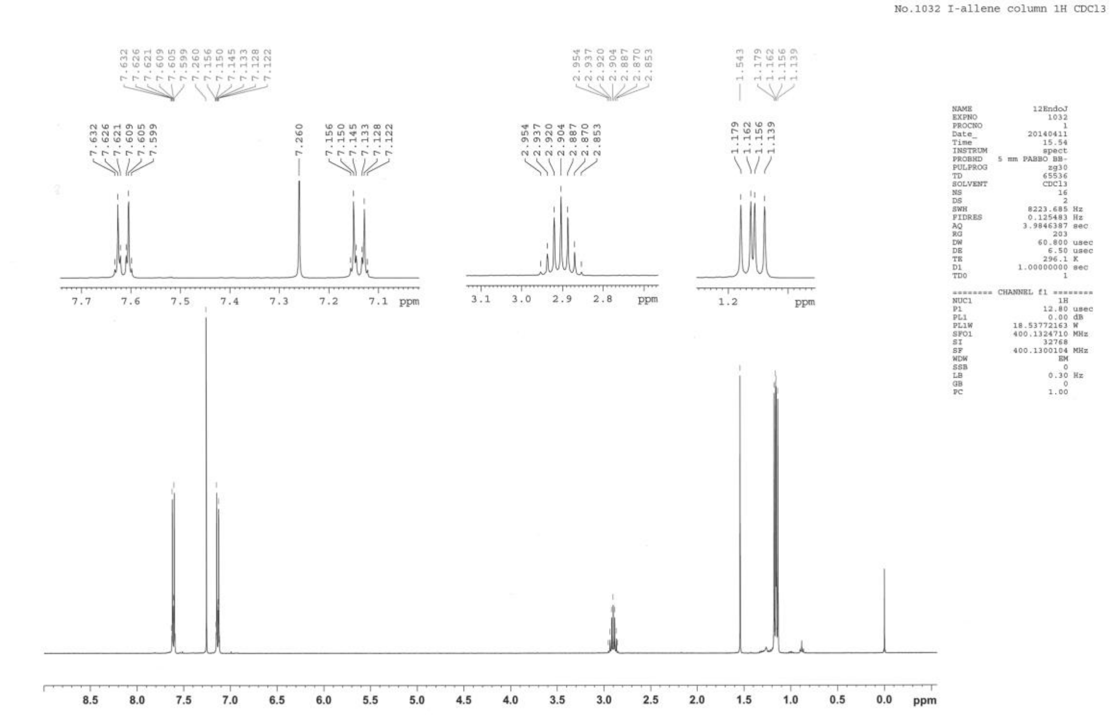


(b) $^{13}$C NMR (100 MHz, $CDCl_3$)

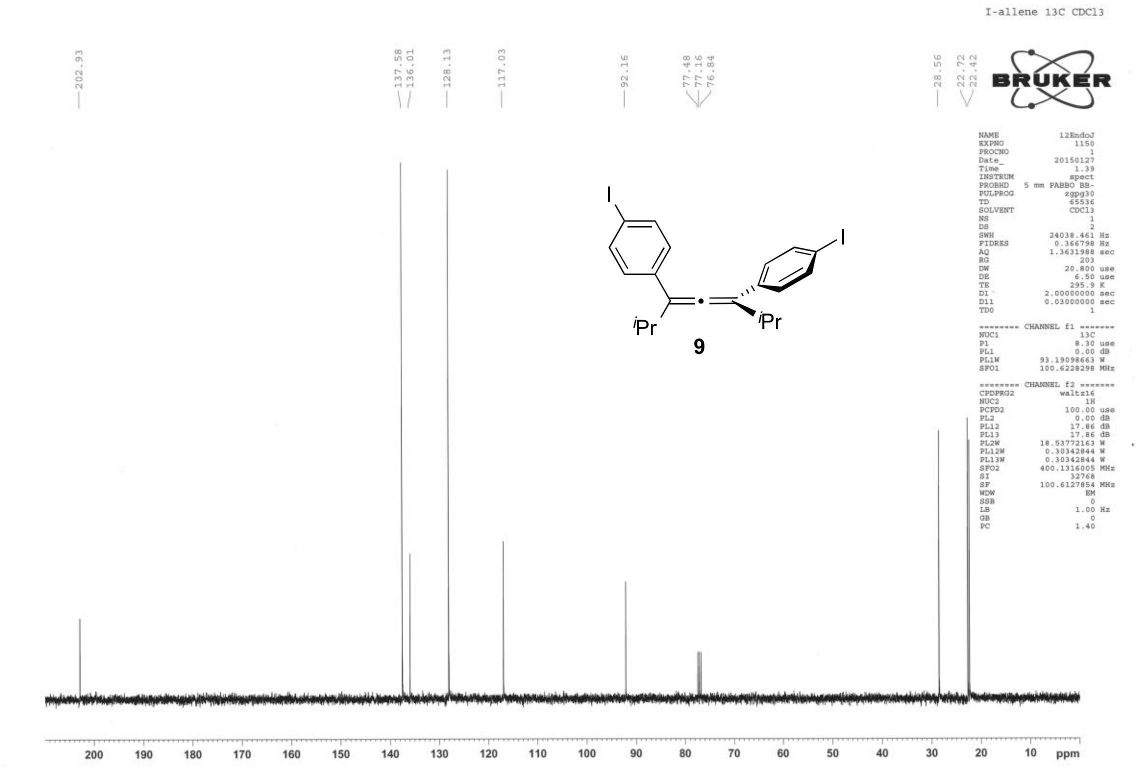

## S8. Figure S6. $^{1}H$ and $^{13}C$ NMR Chart of 3

(a)

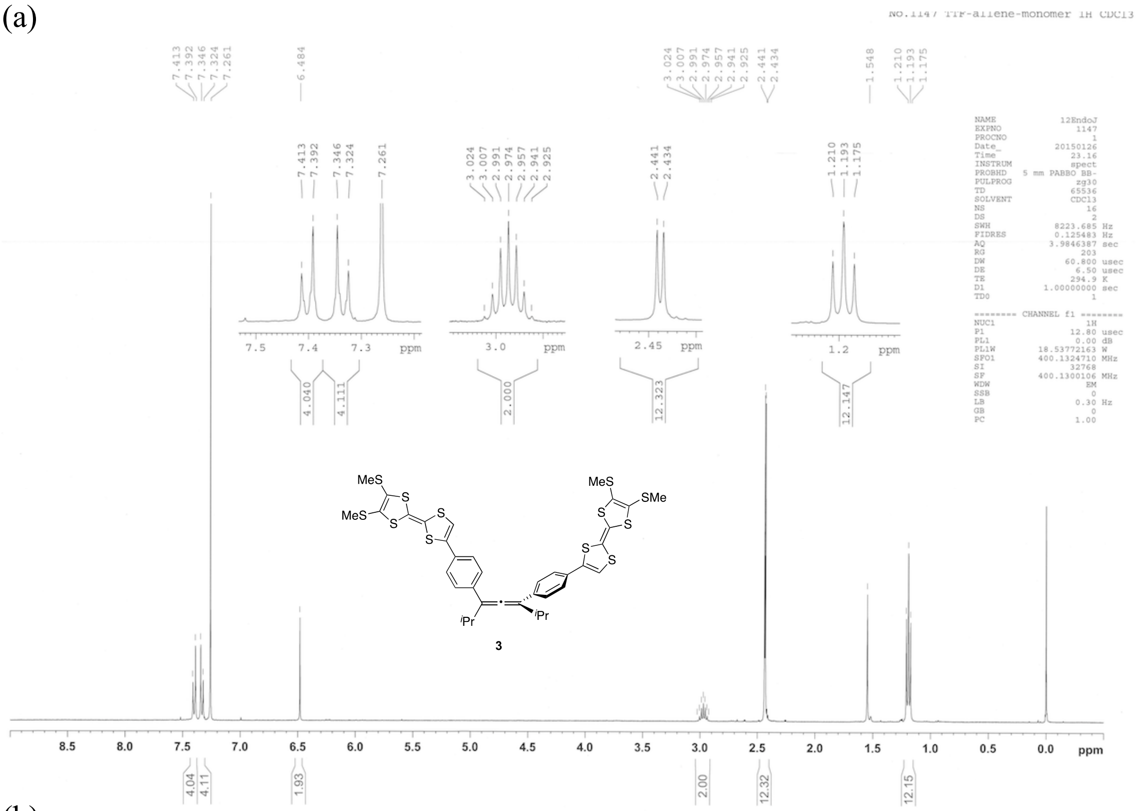

(b)

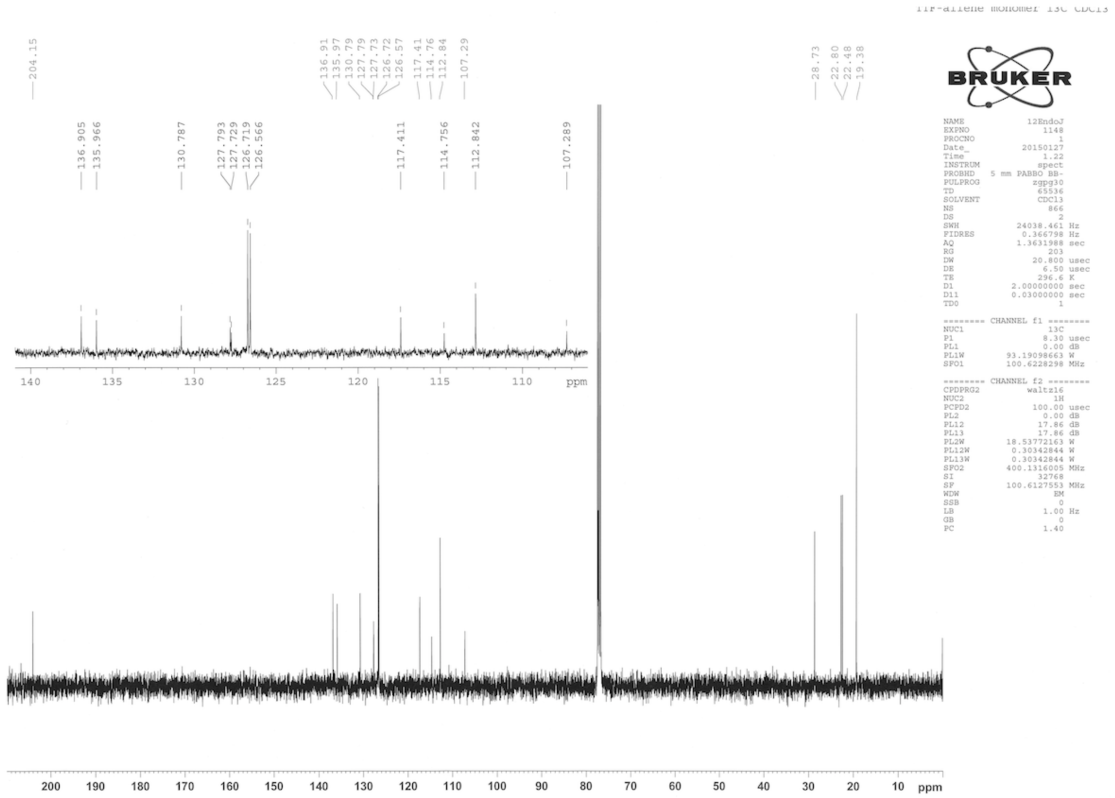

**S9. Figure S7. $^{1}$H NMR Spectrum of (a) (*R*)-PTDPA and (b) (*S*)- PTDPA**

(a)

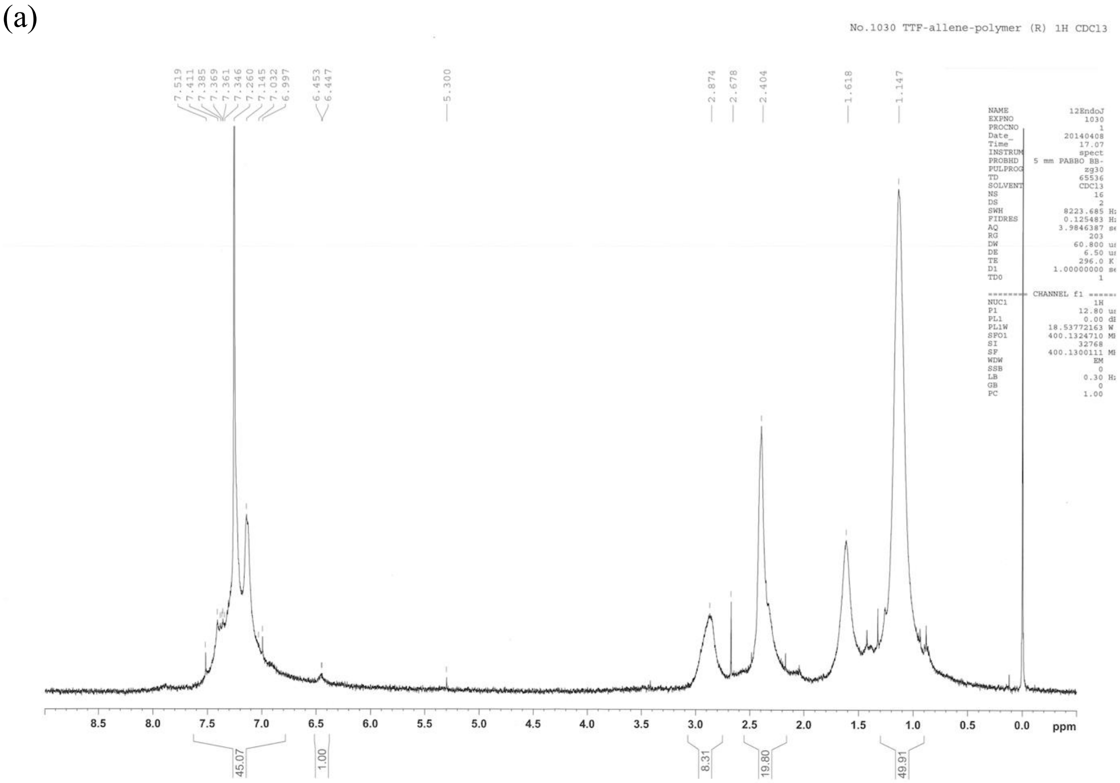


(b)

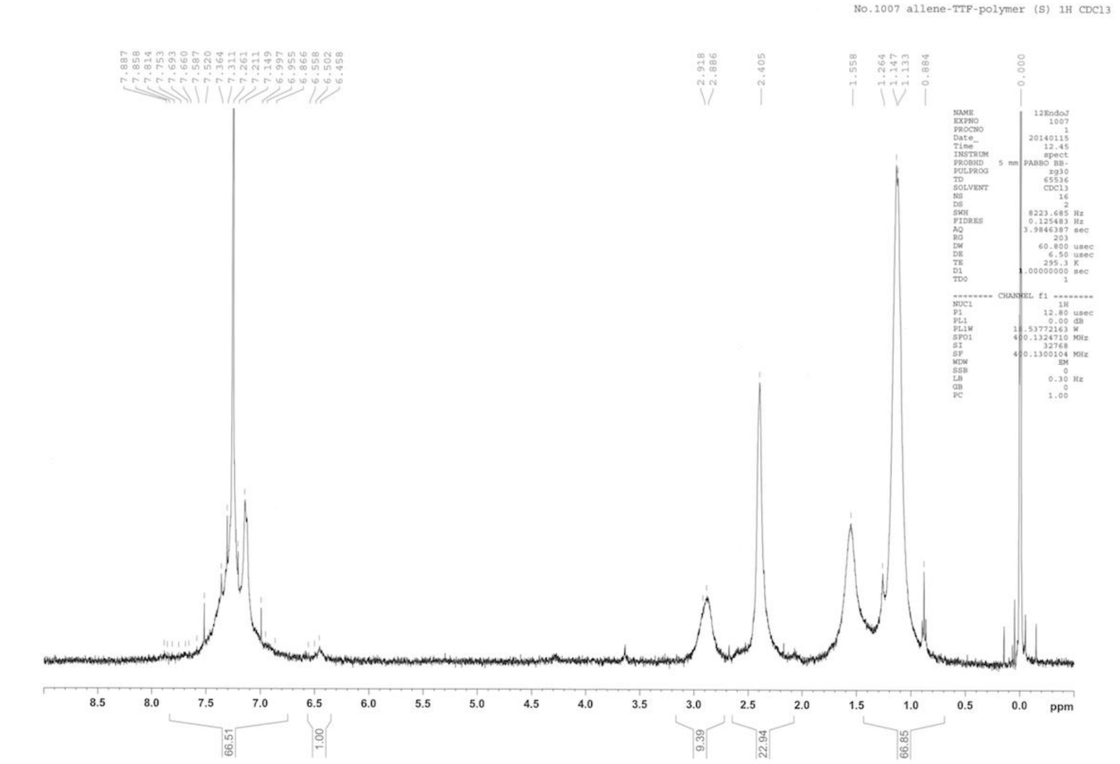

**S10. Figure S8. Chiral HPLC Chart of (a) 9 and (b) 3**

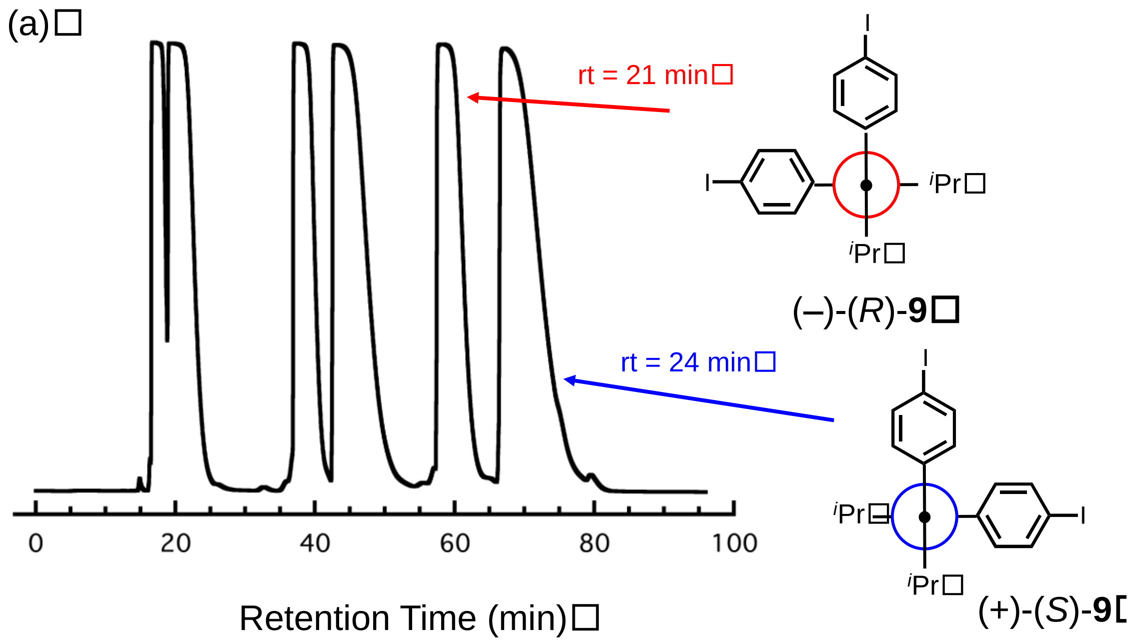


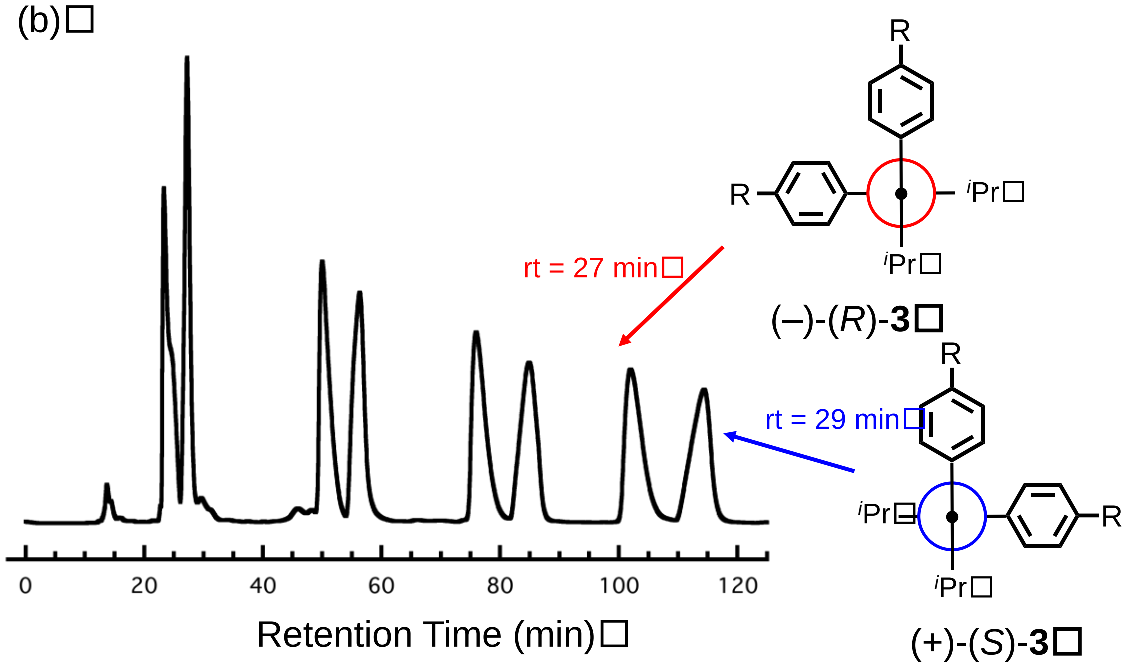

**S11. DFT Calculations of 9 and 3**

(a) DFT calculation of (*R*)-**9**

The geometry optimization was performed by DFT calculation with B3LYP/6-31G(d,p) (for C and H) and LANL2DZ (for I) basis sets. The optimized structure was confirmed by further frequency calculations. The geometry having the lowest energy was treated further TD-B3LYP calculation.

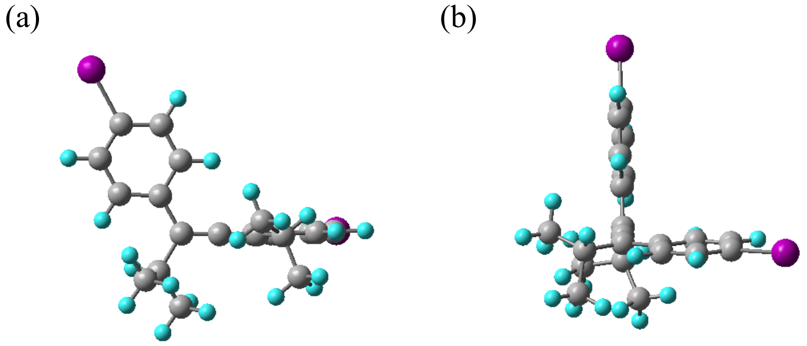


**Figure S10.** Optimized structure of (*R*)-**9**. (a) Top View (b) side view.

**Table S1.** Molecular coordinate of optimized structure of (*R*)-**9**.

| Center Number | Atomic Number | Atomic Type | Coordinates (Angstroms) X | Y | Z |
|---|---|---|---|---|---|
| 1 | 6 | 0 | 0.000000 | 0.000000 | 1.966285 |
| 2 | 6 | 0 | -0.333544 | 1.276477 | 1.952512 |
| 3 | 6 | 0 | 0.354735 | 2.227021 | 1.024030 |
| 4 | 6 | 0 | 0.000000 | 3.583765 | 0.946682 |
| 5 | 1 | 0 | -0.788602 | 3.984553 | 1.572699 |
| 6 | 6 | 0 | 0.642901 | 4.459738 | 0.068207 |
| 7 | 1 | 0 | 0.345146 | 5.501810 | 0.034122 |
| 8 | 6 | 0 | 1.658903 | 3.982320 | -0.752676 |
| 9 | 6 | 0 | 2.034758 | 2.638913 | -0.704588 |
| 10 | 1 | 0 | 2.824023 | 2.261092 | -1.344626 |
| 11 | 6 | 0 | 1.385737 | 1.780362 | 0.175917 |
| 12 | 1 | 0 | 1.679067 | 0.735693 | 0.204972 |
| 13 | 6 | 0 | 0.333544 | -1.276477 | 1.952512 |
| 14 | 6 | 0 | -0.354735 | -2.227021 | 1.024030 |
| 15 | 6 | 0 | 0.000000 | -3.583765 | 0.946682 |
| 16 | 1 | 0 | 0.788602 | -3.984553 | 1.572699 |
| 17 | 6 | 0 | -0.642901 | -4.459738 | 0.068207 |
| 18 | 1 | 0 | -0.345146 | -5.501810 | 0.034122 |

| | | | | | |
|---|---|---|---|---|---|
| 19 | 6 | 0 | -1.658903 | -3.982320 | -0.752676 |
| 20 | 6 | 0 | -2.034758 | -2.638913 | -0.704588 |
| 21 | 1 | 0 | -2.824023 | -2.261092 | -1.344626 |
| 22 | 6 | 0 | -1.385737 | -1.780362 | 0.175917 |
| 23 | 1 | 0 | -1.679067 | -0.735693 | 0.204972 |
| 24 | 53 | 0 | 2.658362 | 5.314920 | -2.101764 |
| 25 | 53 | 0 | -2.658362 | -5.314920 | -2.101764 |
| 26 | 6 | 0 | 1.411537 | -1.804497 | 2.911444 |
| 27 | 1 | 0 | 2.018859 | -2.524190 | 2.347265 |
| 28 | 6 | 0 | -1.411537 | 1.804497 | 2.911444 |
| 29 | 1 | 0 | -2.018859 | 2.524190 | 2.347265 |
| 30 | 6 | 0 | -0.769938 | 2.546742 | 4.102411 |
| 31 | 1 | 0 | -1.540740 | 3.008340 | 4.729062 |
| 32 | 1 | 0 | -0.205252 | 1.844108 | 4.724806 |
| 33 | 1 | 0 | -0.080850 | 3.331501 | 3.778277 |
| 34 | 6 | 0 | -2.362280 | 0.709493 | 3.411875 |
| 35 | 1 | 0 | -1.828311 | -0.041483 | 4.002622 |
| 36 | 1 | 0 | -3.138145 | 1.147108 | 4.048375 |
| 37 | 1 | 0 | -2.853531 | 0.192708 | 2.582202 |
| 38 | 6 | 0 | 2.362280 | -0.709493 | 3.411875 |
| 39 | 1 | 0 | 1.828311 | 0.041483 | 4.002622 |
| 40 | 1 | 0 | 3.138145 | -1.147108 | 4.048375 |
| 41 | 1 | 0 | 2.853531 | -0.192708 | 2.582202 |
| 42 | 6 | 0 | 0.769938 | -2.546742 | 4.102411 |
| 43 | 1 | 0 | 1.540740 | -3.008340 | 4.729062 |
| 44 | 1 | 0 | 0.205252 | -1.844108 | 4.724806 |
| 45 | 1 | 0 | 0.080850 | -3.331501 | 3.778277 |

---------------------------------------------------------------------------------------------------

Name: (*R*)-**9** (file: iodo)

Method: B3LYP/6-31G(d,p) (for C and H) and LAN2DZ (for I)

Key word: opt freq scf=(direct, tight)

Symmetry: $C_2$

# of imaginary frequency: 0

Energy: -836.2298235 Hartrees

(b) DFT calculation of (*R*)-**3**

The geometry optimizations were performed by DFT calculation with B3LYP/6-31G(d,p) basis set. We started with two conformations of (*R*)-**3**-A and (*R*)-**3**-B, which have different orientations of TTF moieties. These conformers were obtained from Z-matrix formatted initial structures. The minimum energy of each conformation

was confirmed by frequency calculations. The geometry having the lowest energy (*R*)-**3**-A was treated further TD-B3LYP calculation.

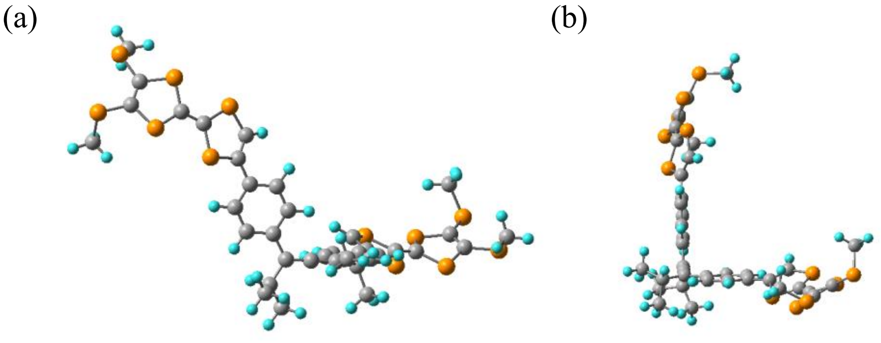

**Figure S11.** Optimized structure of (*R*)-**3**-A. (a) Top View (b) side view.

**Table S2.** Molecular coordinate of optimized structure of (*R*)-**3**-A.

| Center Number | Atomic Number | Atomic Type | Coordinates (Angstroms) X | Y | Z |
|---|---|---|---|---|---|
| 1 | 6 | 0 | 0.000067 | 3.902933 | -0.000003 |
| 2 | 6 | 0 | 1.146859 | 3.897909 | -0.652891 |
| 3 | 6 | 0 | 2.247715 | 2.974710 | -0.241488 |
| 4 | 6 | 0 | 3.461811 | 2.896373 | -0.942808 |
| 5 | 1 | 0 | 3.634078 | 3.516766 | -1.814072 |
| 6 | 6 | 0 | 4.472817 | 2.020656 | -0.552132 |
| 7 | 1 | 0 | 5.387195 | 1.975676 | -1.135755 |
| 8 | 6 | 0 | 4.316534 | 1.180831 | 0.560026 |
| 9 | 6 | 0 | 3.104965 | 1.264427 | 1.274368 |
| 10 | 1 | 0 | 2.964988 | 0.657848 | 2.163731 |
| 11 | 6 | 0 | 2.099874 | 2.131248 | 0.878367 |
| 12 | 1 | 0 | 1.177315 | 2.171396 | 1.448355 |
| 13 | 6 | 0 | -1.146728 | 3.898019 | 0.652877 |
| 14 | 6 | 0 | -2.247670 | 2.974920 | 0.241465 |
| 15 | 6 | 0 | -3.461560 | 2.896291 | 0.943107 |
| 16 | 1 | 0 | -3.633591 | 3.516386 | 1.814630 |
| 17 | 6 | 0 | -4.472635 | 2.020653 | 0.552433 |
| 18 | 1 | 0 | -5.386842 | 1.975426 | 1.136304 |
| 19 | 6 | 0 | -4.316624 | 1.181207 | -0.560050 |
| 20 | 6 | 0 | -3.105270 | 1.265107 | -1.274718 |
| 21 | 1 | 0 | -2.965529 | 0.658838 | -2.164329 |
| 22 | 6 | 0 | -2.100112 | 2.131855 | -0.878726 |

| | | | | | |
|---|---|---|---|---|---|
| 23 | 1 | 0 | -1.177728 | 2.172250 | -1.448978 |
| 24 | 6 | 0 | -1.369751 | 4.863805 | 1.828460 |
| 25 | 1 | 0 | -1.900443 | 4.302139 | 2.608184 |
| 26 | 6 | 0 | 1.369985 | 4.863718 | -1.828441 |
| 27 | 1 | 0 | 1.900860 | 4.302093 | -2.608071 |
| 28 | 6 | 0 | 2.257779 | 6.049970 | -1.401669 |
| 29 | 1 | 0 | 2.498408 | 6.680482 | -2.264428 |
| 30 | 1 | 0 | 1.733003 | 6.670237 | -0.667082 |
| 31 | 1 | 0 | 3.196880 | 5.721156 | -0.948831 |
| 32 | 6 | 0 | 0.061848 | 5.367685 | -2.450604 |
| 33 | 1 | 0 | -0.520103 | 5.953928 | -1.732682 |
| 34 | 1 | 0 | 0.277360 | 6.009213 | -3.311019 |
| 35 | 1 | 0 | -0.567087 | 4.540391 | -2.792170 |
| 36 | 6 | 0 | -0.061567 | 5.367901 | 2.450413 |
| 37 | 1 | 0 | 0.520165 | 5.954295 | 1.732435 |
| 38 | 1 | 0 | -0.277007 | 6.009326 | 3.310925 |
| 39 | 1 | 0 | 0.567548 | 4.540665 | 2.791785 |
| 40 | 6 | 0 | -2.257754 | 6.049943 | 1.401813 |
| 41 | 1 | 0 | -2.498286 | 6.680469 | 2.264589 |
| 42 | 1 | 0 | -1.733193 | 6.670232 | 0.667091 |
| 43 | 1 | 0 | -3.196910 | 5.720991 | 0.949184 |
| 44 | 6 | 0 | -5.371133 | 0.243476 | -0.973237 |
| 45 | 6 | 0 | -5.187935 | -0.933865 | -1.600938 |
| 46 | 6 | 0 | -7.751807 | -0.874889 | -1.168912 |
| 47 | 1 | 0 | -4.216832 | -1.345598 | -1.846507 |
| 48 | 6 | 0 | 5.370983 | 0.243025 | 0.973192 |
| 49 | 6 | 0 | 5.187704 | -0.934502 | 1.600520 |
| 50 | 6 | 0 | 7.751650 | -0.875327 | 1.168984 |
| 51 | 1 | 0 | 4.216567 | -1.346342 | 1.845776 |
| 52 | 16 | 0 | -7.075322 | 0.694997 | -0.672937 |
| 53 | 16 | 0 | -6.587410 | -1.896499 | -2.052034 |
| 54 | 16 | 0 | 6.587126 | -1.897213 | 2.051619 |
| 55 | 16 | 0 | 7.075220 | 0.694697 | 0.673375 |
| 56 | 6 | 0 | 9.028071 | -1.242856 | 0.928382 |
| 57 | 6 | 0 | 11.253882 | -1.594938 | -0.406922 |
| 58 | 6 | 0 | 11.036098 | -2.772858 | 0.228722 |
| 59 | 6 | 0 | -9.028187 | -1.242507 | -0.928225 |
| 60 | 6 | 0 | -11.253840 | -1.595046 | 0.407221 |
| 61 | 6 | 0 | -11.036120 | -2.772758 | -0.228831 |
| 62 | 16 | 0 | 9.737569 | -2.785574 | 1.460383 |
| 63 | 16 | 0 | 10.211995 | -0.222743 | 0.077932 |
| 64 | 16 | 0 | -9.737726 | -2.785066 | -1.460630 |
| 65 | 16 | 0 | -10.212019 | -0.222685 | -0.077302 |
| 66 | 16 | 0 | -12.576225 | -1.259052 | 1.529008 |
| 67 | 16 | 0 | -12.030595 | -4.224150 | -0.074117 |

| | | | | | |
|---|---|---|---|---|---|
| 68 | 16 | 0 | 12.030570 | -4.224215 | 0.073644 |
| 69 | 16 | 0 | 12.576444 | -1.258575 | -1.528394 |
| 70 | 6 | 0 | -10.789614 | -5.473982 | 0.448588 |
| 71 | 1 | 0 | -10.376573 | -5.221878 | 1.426465 |
| 72 | 1 | 0 | -11.334086 | -6.418462 | 0.516424 |
| 73 | 1 | 0 | -9.989450 | -5.570794 | -0.286172 |
| 74 | 6 | 0 | -11.662610 | -0.731019 | 3.033069 |
| 75 | 1 | 0 | -12.428171 | -0.448211 | 3.759026 |
| 76 | 1 | 0 | -11.069409 | -1.554867 | 3.432829 |
| 77 | 1 | 0 | -11.024401 | 0.129272 | 2.827663 |
| 78 | 6 | 0 | 10.789692 | -5.473748 | -0.450034 |
| 79 | 1 | 0 | 10.377050 | -5.221223 | -1.427971 |
| 80 | 1 | 0 | 11.334107 | -6.418250 | -0.518030 |
| 81 | 1 | 0 | 9.989238 | -5.570773 | 0.284381 |
| 82 | 6 | 0 | 11.663111 | -0.729542 | -3.032276 |
| 83 | 1 | 0 | 12.428831 | -0.446649 | -3.758032 |
| 84 | 1 | 0 | 11.069678 | -1.553009 | -3.432475 |
| 85 | 1 | 0 | 11.025169 | 0.130865 | -2.826529 |

---------------------------------------------------------------------------------------------------

Name: (*R*)-**3**-A

Method: B3LYP/6-31G(d,p)

Key word: opt freq scf=(direct, tight)

Symmetry: $C_2$

# of imaginary frequency: 0

Energy: -6209.80702127 Hartrees

(a) (b)

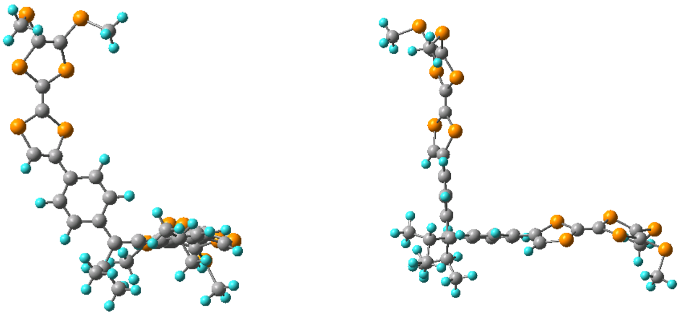

**Figure S12.** Optimized structure of (*R*)-**3**-B. (a) Top View (b) side view.

**Table S3.** Molecular coordinate of optimized structure of (*R*)-**3**-B.

| Center Number | Atomic Number | Atomic Type | Coordinates (Angstroms) X | Y | Z |
|---|---|---|---|---|---|
| 1 | 6 | 0 | 0.000000 | 0.000000 | 4.654233 |
| 2 | 6 | 0 | 1.154784 | -0.638245 | 4.645318 |

| | | | | | |
|---|---|---|---|---|---|
| 3 | 6 | 0 | 1.374170 | -1.793648 | 3.722494 |
| 4 | 6 | 0 | 2.619858 | -2.437200 | 3.612465 |
| 5 | 1 | 0 | 3.464809 | -2.101754 | 4.201937 |
| 6 | 6 | 0 | 2.814065 | -3.499040 | 2.734735 |
| 7 | 1 | 0 | 3.800598 | -3.944350 | 2.651722 |
| 8 | 6 | 0 | 1.766766 | -3.981932 | 1.934602 |
| 9 | 6 | 0 | 0.520987 | -3.334445 | 2.032192 |
| 10 | 1 | 0 | -0.314502 | -3.684098 | 1.433408 |
| 11 | 6 | 0 | 0.333911 | -2.270972 | 2.903253 |
| 12 | 1 | 0 | -0.639009 | -1.793214 | 2.955380 |
| 13 | 6 | 0 | -1.154784 | 0.638245 | 4.645318 |
| 14 | 6 | 0 | -1.374170 | 1.793648 | 3.722494 |
| 15 | 6 | 0 | -2.619858 | 2.437200 | 3.612465 |
| 16 | 1 | 0 | -3.464809 | 2.101754 | 4.201937 |
| 17 | 6 | 0 | -2.814065 | 3.499040 | 2.734735 |
| 18 | 1 | 0 | -3.800598 | 3.944350 | 2.651722 |
| 19 | 6 | 0 | -1.766766 | 3.981932 | 1.934602 |
| 20 | 6 | 0 | -0.520987 | 3.334445 | 2.032192 |
| 21 | 1 | 0 | 0.314502 | 3.684098 | 1.433408 |
| 22 | 6 | 0 | -0.333911 | 2.270972 | 2.903253 |
| 23 | 1 | 0 | 0.639009 | 1.793214 | 2.955380 |
| 24 | 6 | 0 | -2.278259 | 0.217179 | 5.606917 |
| 25 | 1 | 0 | -3.214675 | 0.238712 | 5.034057 |
| 26 | 6 | 0 | 2.278259 | -0.217179 | 5.606917 |
| 27 | 1 | 0 | 3.214675 | -0.238712 | 5.034057 |
| 28 | 6 | 0 | 2.406570 | -1.217909 | 6.772750 |
| 29 | 1 | 0 | 3.271125 | -0.970337 | 7.398166 |
| 30 | 1 | 0 | 1.512583 | -1.181269 | 7.404660 |
| 31 | 1 | 0 | 2.523933 | -2.246877 | 6.422833 |
| 32 | 6 | 0 | 2.112719 | 1.210979 | 6.140767 |
| 33 | 1 | 0 | 1.200290 | 1.310201 | 6.737078 |
| 34 | 1 | 0 | 2.961377 | 1.472740 | 6.780777 |
| 35 | 1 | 0 | 2.060580 | 1.941772 | 5.328588 |
| 36 | 6 | 0 | -2.112719 | -1.210979 | 6.140767 |
| 37 | 1 | 0 | -1.200290 | -1.310201 | 6.737078 |
| 38 | 1 | 0 | -2.961377 | -1.472740 | 6.780777 |
| 39 | 1 | 0 | -2.060580 | -1.941772 | 5.328588 |
| 40 | 6 | 0 | -2.406570 | 1.217909 | 6.772750 |
| 41 | 1 | 0 | -3.271125 | 0.970337 | 7.398166 |
| 42 | 1 | 0 | -1.512583 | 1.181269 | 7.404660 |
| 43 | 1 | 0 | -2.523933 | 2.246877 | 6.422833 |
| 44 | 6 | 0 | -1.973477 | 5.124038 | 1.031270 |
| 45 | 6 | 0 | -2.825621 | 6.148020 | 1.227629 |
| 46 | 6 | 0 | -1.583863 | 6.808703 | -0.960420 |
| 47 | 1 | 0 | -3.440761 | 6.263476 | 2.111462 |

| | | | | | |
|---|---|---|---|---|---|
| 48 | 6 | 0 | 1.973477 | -5.124038 | 1.031270 |
| 49 | 6 | 0 | 2.825621 | -6.148020 | 1.227629 |
| 50 | 6 | 0 | 1.583863 | -6.808703 | -0.960420 |
| 51 | 1 | 0 | 3.440761 | -6.263476 | 2.111462 |
| 52 | 16 | 0 | -1.059373 | 5.169743 | -0.504795 |
| 53 | 16 | 0 | -2.962685 | 7.401245 | 0.002997 |
| 54 | 16 | 0 | 2.962685 | -7.401245 | 0.002997 |
| 55 | 16 | 0 | 1.059373 | -5.169743 | -0.504795 |
| 56 | 6 | 0 | 1.015953 | -7.520070 | -1.957172 |
| 57 | 6 | 0 | -0.873475 | -8.571703 | -3.435574 |
| 58 | 6 | 0 | 0.000000 | -9.584246 | -3.210432 |
| 59 | 6 | 0 | -1.015953 | 7.520070 | -1.957172 |
| 60 | 6 | 0 | 0.873475 | 8.571703 | -3.435574 |
| 61 | 6 | 0 | 0.000000 | 9.584246 | -3.210432 |
| 62 | 16 | 0 | 1.562721 | -9.136652 | -2.461749 |
| 63 | 16 | 0 | -0.338050 | -6.933815 | -2.951552 |
| 64 | 16 | 0 | -1.562721 | 9.136652 | -2.461749 |
| 65 | 16 | 0 | 0.338050 | 6.933815 | -2.951552 |
| 66 | 16 | 0 | 2.399392 | 8.704883 | -4.314987 |
| 67 | 16 | 0 | 0.199725 | 11.253818 | -3.750992 |
| 68 | 16 | 0 | -0.199725 | -11.253818 | -3.750992 |
| 69 | 16 | 0 | -2.399392 | -8.704883 | -4.314987 |
| 70 | 6 | 0 | 0.052394 | 12.179170 | -2.170784 |
| 71 | 1 | 0 | 0.868340 | 11.920274 | -1.494299 |
| 72 | 1 | 0 | 0.125218 | 13.235059 | -2.440891 |
| 73 | 1 | 0 | -0.909334 | 11.995254 | -1.690452 |
| 74 | 6 | 0 | 3.603324 | 8.106826 | -3.062473 |
| 75 | 1 | 0 | 4.574476 | 8.108361 | -3.562405 |
| 76 | 1 | 0 | 3.634633 | 8.781253 | -2.205384 |
| 77 | 1 | 0 | 3.366379 | 7.093061 | -2.737299 |
| 78 | 6 | 0 | -0.052394 | -12.179170 | -2.170784 |
| 79 | 1 | 0 | -0.868340 | -11.920274 | -1.494299 |
| 80 | 1 | 0 | -0.125218 | -13.235059 | -2.440891 |
| 81 | 1 | 0 | 0.909334 | -11.995254 | -1.690452 |
| 82 | 6 | 0 | -3.603324 | -8.106826 | -3.062473 |
| 83 | 1 | 0 | -4.574476 | -8.108361 | -3.562405 |
| 84 | 1 | 0 | -3.634633 | -8.781253 | -2.205384 |

| | | | | | |
|---|---|---|---|---|---|
| 85 | 1 | 0 | -3.366379 | -7.093061 | -2.737299 |

---------------------------------------------------------------------------------------------------

Name: (*R*)-**3**-B

Method: B3LYP/6-31G(d,p)

Key word: opt freq scf=(direct, tight)

Symmetry: $C_2$

# of imaginary frequency: 0

Energy: -6209.80655334 Hartrees

## S12. TD-DFT Calculations and MO diagram of 9 and 3

(a) Compound **9**

**Table S4.** Selected electronic transition for (*R*)-**9**

| excited state | energy | oscillator strengths | Rotational Strength in cgs ($10^{-40}$esu$^2$cm$^2$) | Nature | |
|---|---|---|---|---|---|
| $S_1$ | 278 nm | 1.0388 | -394.472 | 80 ->82 | 0.47828 |
| | 4.459 eV | | | 81 ->83 | 0.49995 |
| $S_2$ | 264 nm | 0.0877 | 174.519 | 80 ->83 | 0.38091 |
| | 4.704 eV | | | 81 ->82 | 0.37065 |
| $S_3$ | 253 nm | 0.1599 | 133.333 | 80->83 | -0.30825 |
| | 4.907 eV | | | 80->85 | -0.30371 |

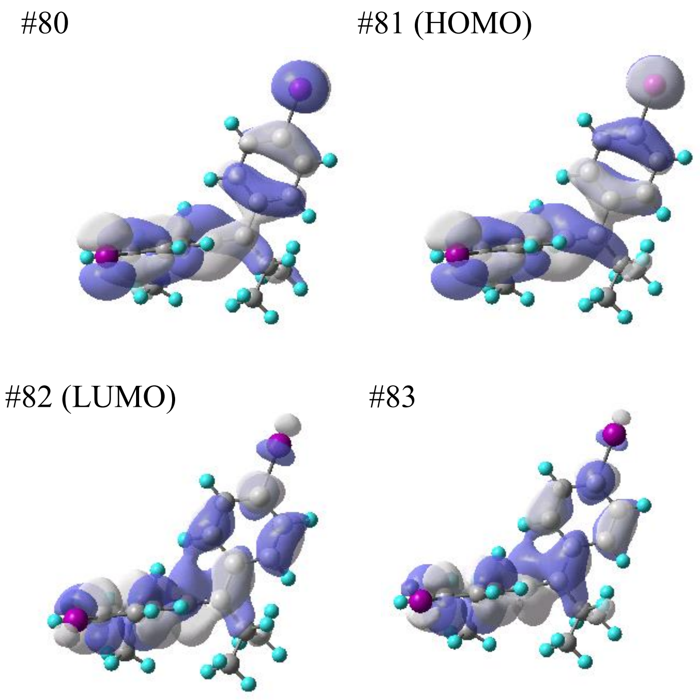


**Figure S13.** MO diagram of (*R*)-**9**.

(b) Compound (*R*)-**3**-A

**Table S5.** Selected electronic transition for (*R*)-**3**

| excited state | energy | oscillator strengths | Rotational Strength in cgs ($10^{-40}$esu$^2$cm$^2$) | Nature | |
|---|---|---|---|---|---|
| $S_1$ | 452 nm<br>2.745 eV | 0.0878 | -36.3764 | 224 ->226<br>225 ->227 | 0.40915<br>0.3678 |
| $S_2$ | 426 nm<br>2.909 eV | 0.1832 | -75.8371 | 224 ->229<br>225 ->228 | 0.39293<br>0.39384 |
| $S_3$ | 372 nm<br>3.331 eV | 0.0415 | 109.017 | 224 ->231<br>225 ->230 | 0.48046<br>0.48110 |
| $S_4$ | 330 nm<br>3.753 eV | 0.4030 | 138.119 | 224 ->233<br>225 ->232 | 0.39970<br>0.44770 |
| $S_5$ | 329 nm<br>3.769 eV | 0.0428 | -123.679 | 224 ->232<br>225 ->233 | 0.47300<br>0.41671 |
| $S_6$ | 317 nm<br>3.915 eV | 0.6504 | 238.909 | 222 ->227 | 0.52053 |

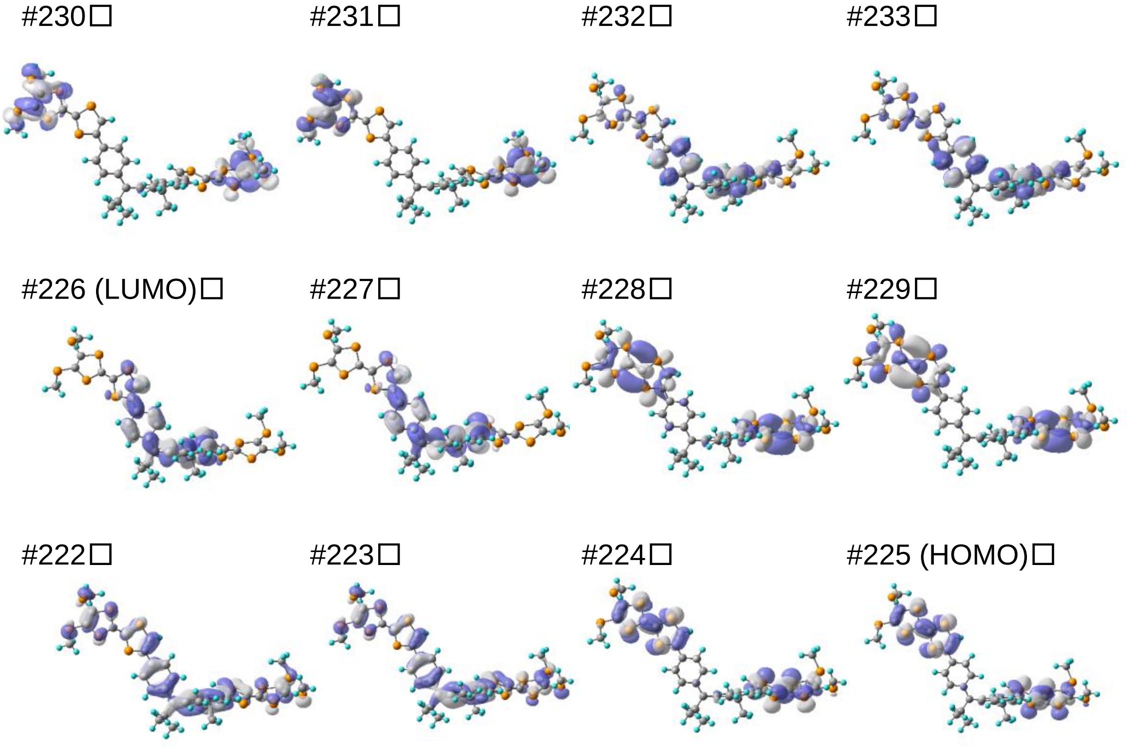


**Figure S14.** MO diagram of (*R*)-**3**.

## S13. ECD Spectra of Cationic Species of (*R*)-3 and (*R*)-PTDTA

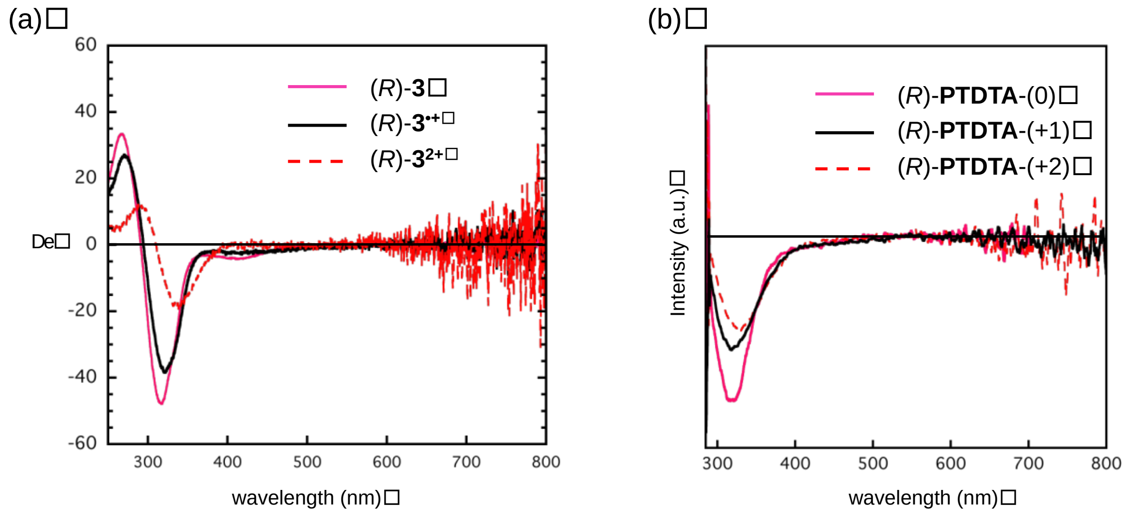


**Figure S15.** ECD Spectra of (a) (*R*)-**3** and its cation radicals, and (b) (*R*)-**PTDTA** and its cationic states.

## S14. Examination of the Photoracemization

Racemization behavior was examined under daylight in $CH_2Cl_2$ solution. The photoracemization was evaluated from the decrease of the ellipticity at 340 nm (for (*R*)-**1**, 320 nm (for (*R*)-**3**), and 250 nm (for (*R*)-**PTDTA**). Data of (*R*)-**1** was obtained from ref. 10. Consequently, the ellipticity of (*R*)-**3** and (*R*)-**PTDTA** almost did not change under ambient light.

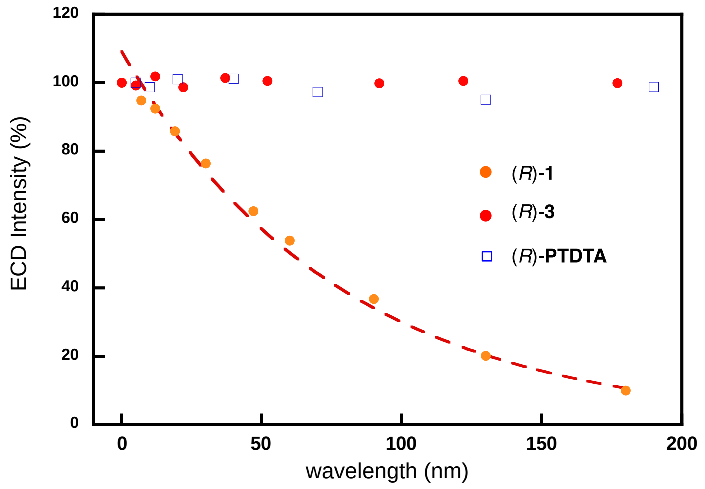


**Figure S16.** Relative ellipticity of (*R*)-**1**, (*R*)-**3**, and (*R*)- **PTDTA** in ECD spectra (in $CH_2Cl_2$, 25°C).

## S15. References

S1. M. Hatano, S. Suzuki, K. Ishihara, *Synlett*, **2009**, 321.
